# Transcriptomic Analysis Reveals Candidate Genes Responding Maize Gray Leaf Spot Caused by *Cercospora zeina*

**DOI:** 10.3390/plants10112257

**Published:** 2021-10-22

**Authors:** Wenzhu He, Yonghui Zhu, Yifeng Leng, Lin Yang, Biao Zhang, Junpin Yang, Xiao Zhang, Hai Lan, Haitao Tang, Jie Chen, Shibin Gao, Jun Tan, Jiwei Kang, Luchang Deng, Yan Li, Yuanyuan He, Tingzhao Rong, Moju Cao

**Affiliations:** 1 Crop Research Institute, Sichuan Academy of Agricultural Sciences, Chengdu 610066, China; yhzhu86@hotmail.com (Y.Z.); ownmylife@163.com (L.Y.); 13908177069@163.com (B.Z.); y.junpin@263.net (J.Y.); 13808068781@163.com (H.T.); 18010503015@163.com (J.C.); tanjunbreeze@163.com (J.T.); kjwkd@163.com (J.K.); leo20060608@126.com (L.D.); zhangxiaoliyan@163.com (Y.L.); heyuan199301@163.com (Y.H.); 2 Maize Research Institute, Sichuan Agricultural University, Chengdu 611130, China; hunterzap@163.com (X.Z.); lanhai_maize@163.com (H.L.); shibingao@163.com (S.G.); rongtz@sicau.edu.cn (T.R.); 3 College of Agricultural Sciences, Xichang University, Xichang 615000, China; yifeng_71@163.com

**Keywords:** maize, RNA-Seq, gray leaf spot, WGCNA, disease resistance

## Abstract

Gray leaf spot (GLS), caused by the fungal pathogen *Cercospora zeina* (*C. zeina*), is one of the most destructive soil-borne diseases in maize (*Zea mays* L.), and severely reduces maize production in Southwest China. However, the mechanism of resistance to GLS is not clear and few resistant alleles have been identified. Two maize inbred lines, which were shown to be resistant (R6) and susceptible (S8) to GLS, were injected by *C. zeina* spore suspensions. Transcriptome analysis was carried out with leaf tissue at 0, 6, 24, 144, and 240 h after inoculation. Compared with 0 h of inoculation, a total of 667 and 419 stable common differentially expressed genes (DEGs) were found in the resistant and susceptible lines across the four timepoints, respectively. The DEGs were usually enriched in ‘response to stimulus’ and ‘response to stress’ in GO term analysis, and ‘plant–pathogen interaction’, ‘MAPK signaling pathways’, and ‘plant hormone signal transduction’ pathways, which were related to maize’s response to GLS, were enriched in KEGG analysis. Weighted-Genes Co-expression Network Analysis (WGCNA) identified two modules, while twenty hub genes identified from these indicated that plant hormone signaling, calcium signaling pathways, and transcription factors played a central role in GLS sensing and response. Combing DEGs and QTL mapping, five genes were identified as the consensus genes for the resistance of GLS. Two genes, were both putative Leucine-rich repeat protein kinase family proteins, specifically expressed in R6. In summary, our results can provide resources for gene mining and exploring the mechanism of resistance to GLS in maize.

## 1. Introduction

Gray leaf spot (GLS) in maize, *Cercospora zeina* (*C. zeina*), is one of the most destructive foliar diseases. High humidity and mild temperature are favorable conditions for GLS infection [1]. *Cercospora* spores predominantly overwinter in diseased plant debris that remains on the soil surface [2], and conidia produced by the fungus are disseminated onto corn plants by wind and rain splash [3]. The mature GLS lesions run parallel to leaf veins with rectangular spots ranging in color from gray to tan. They first appear as small tan spots at the bottom leaf, and as the disease develops, the entire leaves and stem become withered. The disease causes substantial losses in yield (from 20% to 60%, and even as high as 100%) in severe cases [4,5,6].

The main methods of controlling GLS include avoiding the conservation of tillage, the application of chemical fungicides, and planting resistant hybrids [7,8]. As conserving tillage is important for preserving the economics of maize production, and chemical fungicides are not environmentally friendly, the selection of resistant hybrids is the most efficient and cost-effective method of controlling GLS [8,9].

GLS resistance is a complex phenotype that is influenced by environmental conditions and controlled by many minor quantitative trait loci (QTL) with additive effects [10,11,12,13]. In the last 20 years, many QTLs related to GLS resistance have been detected on all 10 maize chromosomes using various mapping populations [6,10,11,13,14,15,16,17,18,19,20]. Some of them can explain a total phenotypic variation of larger than 20% [15,20]. Combining genome-wide association and linkage mapping, Kibe et al. [18] identified 14 QTLs across three DH populations, and 10 significant associated SNPs. Nine of the co-located candidate genes play roles in plant defense against pathogens. However, only a few of the primarily mapped QTL-derived genes have been finely mapped at present. *qRgls2*, which can increase the resistance percentage by more than 20%, were mapped in 1-Mb intervals, and 15 genes were predicted [21]. *Qgls8* was detected by a near-isogenic line population derived from teosinte introgression, and delimited in a ~130 kb region on chromosome 8, including five predicted genes [22]. *qGLS1.02*, which can explain 6.86–36.24% of the phenotypic variation in GLS resistance, was narrowed to ~314 kb, including 12 candidate genes [23]. *Qgls_YZ2-1*, which was identified from two DH populations, was finely mapped into a 2.4-Mb region on chromosome 2 [19]. *qRgls1.06*, which was a major QTL that explained ~55% of phenotype variance, was localized in a 2.38-Mb region on chromosome 1 [24]. In summary, despite the abundant GLS QTLs that were identified by the various mapping populations, only few GLS-resistant QTLs were finely mapped, and no GLS-resistant gene has been cloned to date. Thus, the mechanism of resistance to GLS has yet to be fully understood. Combining linkage mapping and omics data is a good strategy to identify the candidate genes related to GLS resistance.

Plants have evolved two types of defense mechanisms against pathogen invasion, i.e., PAMP-triggered immunity (PTI) and effector-triggered immunity (ETI) [25]. Many of the key enzymes are involved in two mechanisms that play roles in plant defense. The phenylalanine ammonia-lysases (PALs) are key enzymes for the biosynthesis of salicylic acid (SA) and the phenylpropanoid pathway of higher plants. The SA is a plant hormone that is required to initiate systemic acquired resistance [26]. The products of the phenylpropanoid pathway are key contributors to disease resistance [27]. PALs contribute to broad-spectrum resistance (BSR) mediated by nucleotide-binding domain, leucine-rich repeat-containing (NLR) proteins and RNA-binding proteins [28,29]. Catalase (CAT) can degrade hydrogen peroxide, which works as a kind of signaling molecule for plant defense in plants [30,31], into water and molecular oxygen. Polyphenol oxidase (POD) is involved in the response to both biotic and abiotic stresses in plants, including plant defense, plastidic oxygen, and the phenylpropanoid pathway [32]. Proline is a widely distributed compatible solutes in plants, and acts as antioxidative defense molecule and signaling molecule during stress [33]. Therefore, the dynamic changes in physiological characteristics caused by these enzymes and components can reflect the intensity of response to the *C. zeina* infection, acting as indicator when dividing the GLS-resistance or susceptible inbred lines.

In recent years, as transcriptome analysis can provide an efficient way of assessing variation in the global expression of genes, RNA-Seq analysis has been used to study plant–pathogen interactions [34,35,36,37]. RNA-Seq analysis, from a previous study of GLS, showed that different forms of kauralexins were induced by *C. zeina* in both resistant and susceptible sub-tropical maize lines adapted to the climate of southern Africa [38]. Several candidate genes related to resistance to gibberellic ear rot were found by RNA-Seq analysis from global gene expression profiles [39]. By combining the RNA-seq and proteome analyses of two maize cultivars, calmodulin-like protein and leucine-rich repeat receptor-like protein kinase, were found to be candidate proteins, potentially linked with resistance to GLS [40]. Although vast progress has been made in the characterization of mechanisms underlying the defense against *C. zeina*, the molecular mechanism of maize resistance to GLS remains obscure. Therefore, understanding the responses of maize after *C. zeina* infection is important in GLS-resistance breeding.

In the present study, based on the response dynamics of PAL, CAT, POD, and FP after *C. zeina* injection, high-throughput RNA sequencing technology (RNA-Seq) was performed at five representative timepoints on two elite maize inbred lines, R6 and S8, from Southwest China, (previously identified as resistant and susceptible to GLS, respectively). Differentially expressed genes (DEGs) were identified and characterized for function prediction. WGCNA was also analyzed for the construction of a co-expression network and exploitation of the hub genes of the modules related to GLS resistance. Combining these with GLS-resistant QTLs from the previous study, the consensus genes located in the QTL interval were considered candidate genes, which probably play a role in the response to *C. zeina* infection in maize.

## 2. Results

### 2.1. Phenotypic Evaluation in Response to C. zeina Infection

**Figure 1:**
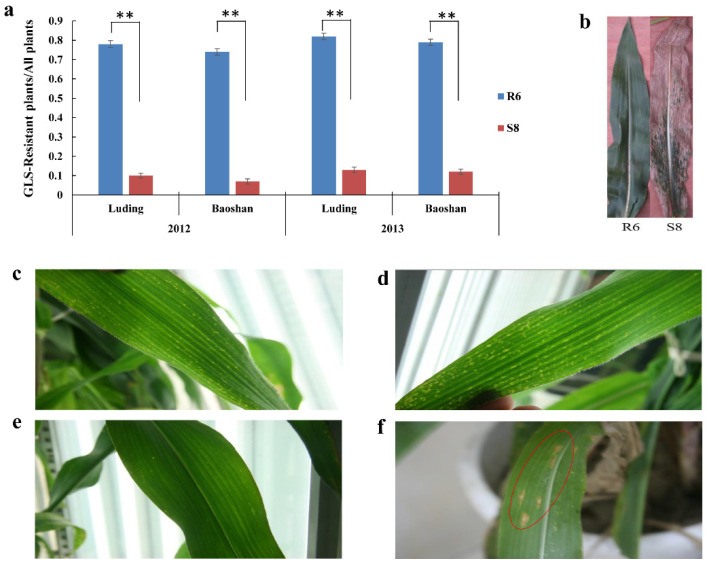
Disease resistance and symptoms of maize inbred lines R6 and S8 in response to *C. zeina*. (**a**) The disease resistance, here also known as GLS resistance, was evaluated 15 days after silking. Asterisks indicate significant differences between R6 and S8 (two-tailed Student’s *t*-test; **: *p* < 0.01). (**b**) Leaves of field-grown R6 and S8 infected by C. *zeina*. (**c**,**d**) R6 leaves’ performance before (**c**) and after (**d**) inoculation with C. *zeina*; (**e**,**f**) S8 leaves before (**e**) and after (**f**) inoculation with *C. zeina*. The infected area is indicated by a red ellipse.

Two inbred maize lines, R6 and S8, were evaluated for quantitative resistance to GLS disease across two environments (Luding and Baoshan) in 2012 and 2013, 15 days after silking. As shown in Figure 1a,b, R6 is significantly resistant to GLS, while S8 is susceptible to GLS. We also assessed the growth status of leaves before and after inoculation with *C. zeina* at the silking stage in a growth chamber. The gray spots on the leaves of S8 were evident after inoculation compared to the control (Figure 1e,f). In contrast, there were no GLS disease symptoms (except small chlorotic spots) on R6 leaves compared to the control (Figure 1c,d).

### 2.2. Dynamic Changes of Physiological Characteristics of Maize Leaves in Response to Inoculation C. zeina

**Figure 2:**
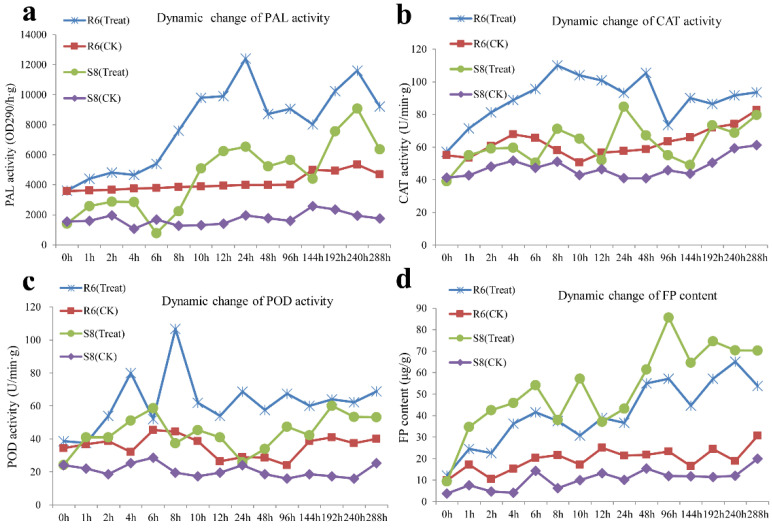
Determination of PAL (**a**), CAT (**b**) and POD (**c**) enzyme activity and FP (**d**) content in maize leaves in response to *C. zeina*. The x axis represents infection at different times after inoculation. Maize inbred lines differing in GLS resistance (R6 = resistant and S8 = sensitive) were inoculated with *C. zeina* (Treat) or not (CK).

The dynamics of physiological indices can explain the degree of response under abiotic and biotic stresses. To identify potential changes in gene expression in response to inoculation by *C. zeina*, the activities of PAL, CAT, POD, and contents of FP in maize leaves were analyzed at continuous stages after *C. zeina* inoculation (Figure 2).

As shown in Figure 2a, the PAL, CAT, POD activity and FP content in R6 was always higher than that in S8 at all timepoints. After C. *zeina* inoculation, the PAL activity in R6 was significantly increased at 6 h, peaked at 24 h, then dropped down. From 144 h PAL activity recovers, increasing and rising to another peak at 240 h. In S8, the PAL activity was significantly decreased at 6 h compared to 0 h; afterwards, it showed a similar trend to R6. The changes in CAT activity in S8 were similar to those of PAL activity, which showed significant decreases at 6 h, 12 h, 144 h, and reached a maximum at 24 h. In R6, from 0 h to 8 h, the CAT activity was increasing and peaked at 8 h; then, it decreased. After 24 h, activity increased again and reached a peak at 48 h, then significant decreases occurred at 96 h. The CAT activity increased slowly from 144 h to 288 h (Figure 2b). The POD activity reached a peak at 8 h and decreased to its lowest 6 h after infection in R6; after 12 h, it changed slightly. In treated S8, POD activity showed a significant increase at 6 h, 96 h, and 192 h and a decrease at 8 h, 24 h, and 144 h (Figure 2c). Most interestingly, as shown in Figure 2b, during treatment, the FP content of S8 was higher than in R6 at almost all timepoints, S8 showed four peaks at 6 h, 10 h, 96 h, and 192 h, then decreased at 8 h, 12 h and 144 h. R6 showed a significant increase at 6 h, 12 h, 96 h, and 240 h, and a significant decrease at 2 h, 10 h, 24 h, and 144 h. However, the PAL, CAT, and POD activities were always higher in R6 than S8.

These results implied that the activities of PAL, CAT, POD, as well as FP content, may participate in the response to GLS in maize, with particularly important changes occurring at 6, 24, 144, and 240 h after inoculation.

### 2.3. Profiling the Maize Leaf Transcriptome Responses to C. zeina Infection

A global gene expression profile of R6 and S8 during *C. zeina* infection was determined in leaves at 0 h, 6 h, 24 h, 144 h, and 240 h post-inoculation. After filtering low-quality reads, a total of 550,290,438 clean reads were obtained and mapped to the reference genome B73 RefGen-V3. About 69% (381,417,240/550,290,438) of the reads were mapped to the B73 genome, of which 97% (371,518,422/381,417,240) were uniquely mapped, including 25,264 and 27,529 genes in the two biological replicates (Table S1). FPKM of expression data revealed a high Pearson’s correlation between the biological replicates (>0.86) for all the analyzed samples, indicating high reproducibility in the sequencing data (Figure S1).

**Figure 3:**
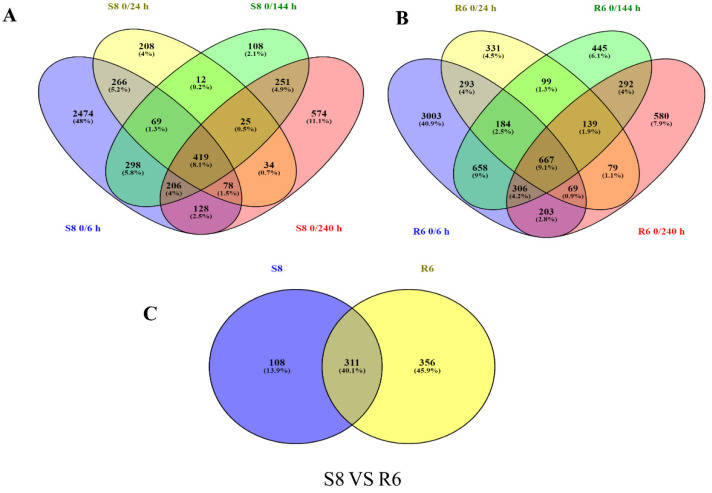
Differentially expressed genes in R6 and S8 at various times after inoculation with *C. zeina*. (**A**) Venn diagrams of DEGs in the GLS-sensitive line S8; (**B**) Venn diagrams of DEGs in resistant line R6. (**C**) Venn diagrams of common DEGs to R6 and S8.

**Figure 4:**
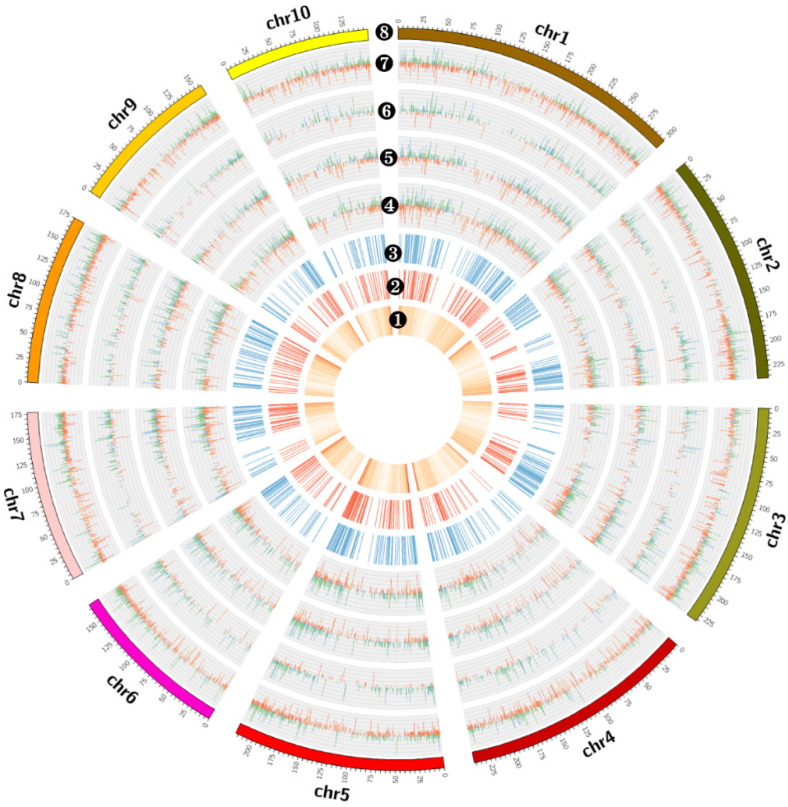
Circular plot of DEG distribution of two inbred lines. The circular plots from the inner to outer layer are ❶ Gene frequency in 1-Mb windows of the whole genome, ❷ Common DEG frequency in multiple timepoints of S8 in 1-Mb windows of the whole genome, ❸ Common DEG frequency in multiple timepoints of R6 1-Mb windows of the whole genome, ❹ log2FC (fold change) of DEGs identified from two inbred lines in 240 h. ❺ log2FC of DEGs identified from two inbred lines in 144 h. ❻ log2FC of DEGs identified from two inbred lines in 24 h. ❼ log2FC of DEGs identified from two inbred lines in 6 h. ❽ Chromosome length. In ❹–❼, the red bars are log2FC of up-regulated DEGs from R6, the blue bars are log2FC of downregulated DEGs from R6, the orange bars are log2FC of upregulated DEGs from S8, the green bars are log2FC of downregulated DEGs from S8.

To characterize the transcriptional changes induced by *C. zeina* infection in -resistant and -susceptible genotypes, we investigated the DEGs of two inbred lines from multiple timepoints after infection (Figure S2). The FPKMs of genes in two inbred lines were controls for DEG analysis compared with 0 h. Hereafter, the 0 h of the S8 sample is named CK1, and the 0 h of the R6 sample is named CK2. In S8, we identified 3938, 1111, 1388, 1715 DEGs in the four timepoints of 6 h, 24 h, 144 h, and 240 h, respectively, and 419 DEGs were consistently detected in these four timepoints (Figure 3A and Figure 4). In R6, 5383, 1861, 2790, and 2335 DEGs were identified in the four times of 6 h, 24 h, 144 h, and 240 h, respectively, with 667 DEGs consistently being detected in all four timepoints (Figure 3B and Figure 4). In DEGs detected in all four timepoints (667 in R6 and 419 in S8), we identified 311 DEGs in both R6 and S8, 356 DEGs in R6, and 108 specific DEGs in S8 (Figure 3C and Figure 4, Table S2).

### 2.4. Real-Time Quantitative Reverse Transcription-PCR (qRT-PCR) Validation

To evaluate the accuracy of the gene expression level calculated by Illumina RNA-Seq, seven DEGs were randomly selected for the real-time quantitative reverse transcription-PCR (qRT-PCR), according to their expression patterns at the three selected timepoints. The expression patterns of those seven DEGs showed consistent trends using the two methods (qRT-PCR and RNA-sequencing) (Figure S3), indicating the accuracy of the applied RNA-seq analysis for studying *C. zeina*-infection-induced maize leaf transcriptome.

### 2.5. Gene Ontology and Pathway Enrichment Analyses of DEGs between R6 and S8

**Figure 5:**
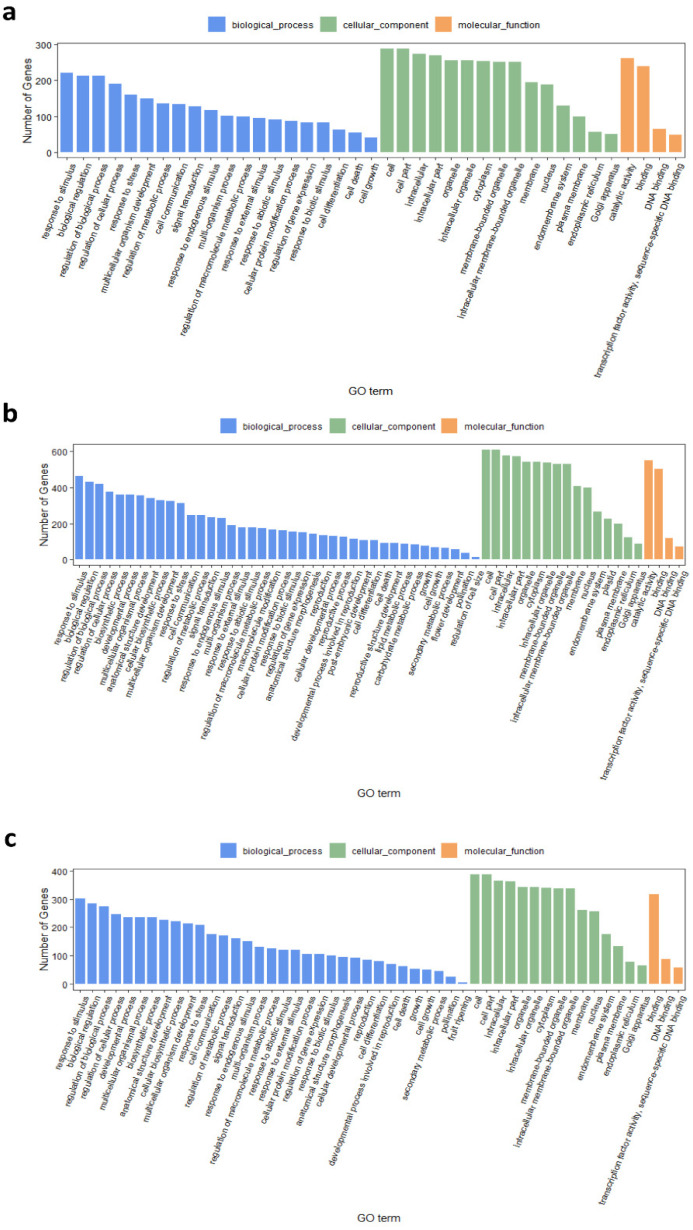
GO term enrichment of common DEGs from R6 and S8 at multiple timepoints. (**a**) GO term enrichment of overlap common DEGs between R6 and S8. (**b**) GO term enrichment of specific common DEGs from R6. (**c**) GO term enrichment of specific common DEGs from S8.

To characterize the function of the DEGs detected in the two inbred lines, GO term enrichment analysis was performed between R6 and S8 (http://systemsbiology.cau.edu.cn/agriGOv2/index.php, accessed on 15 February 2021) with a threshold of FDR < 0.01. The 311 overlapped DEGs were enriched in ‘response to external stimulus’, ‘response to biotic stimulus’, and ‘response to stress’, which were closely related to disease resistance (Figure 5a) in the biological process. Both the specific common DEGs of R6 and S8 were enriched in ‘response to biotic stimulus’, ‘response to external stimulus’, and ‘response to stress’ (Figure 5b,c). To understand the specific function of DEGs in each timepoint, specific DEGs were collected at each timepoint for GO analysis. For R6, the GO terms of the biological process were also enriched in ‘response to abiotic stimulus’, and ‘response to stress’, which were related to disease resistance (Figure S4). For S8, GO terms of the biological process were enriched in ‘response to abiotic stimulus’, ‘response to stress’, ‘response to endogenous stimulus’, ‘response to biotic stimulus’, and ‘response to external stimulus’ (Figure S5). Due to the resistance variance between R6 and S8, we also compared the GO terms at each timepoint between the two inbred lines. It was worth noting that, at four timepoints, specific DEGs of two inbred lines were both enriched in ‘response to stimulus’, and ‘response to biotic stimulus’ in the biological process (Figures S6 and S7), while more GO terms related to reproduction and development were found. This indicated that R6 might maintain the development and reproduction process after *C. zeina* infection. This could be a factor in R6’s increased resistance to gray leaf spot disease.

**Figure 6:**
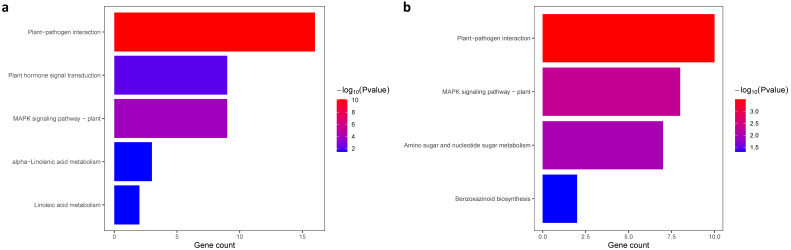
KEGG pathway enrichment of common DEGs from R6 and S8 at multiple timepoints. (**a**) KEGG pathway enrichment of overlap common DEGs between R6 and S8. (**b**) KEGG pathway enrichment of specific common DEGs from R6.

The KEGG pathway analysis (http://kobas.cbi.pku.edu.cn/kobas3/genelist/, accessed on 15 February 2021) was used to elucidate the biological function of specific DEGs with a corrected *p*-valve < 0.05. The 311 common DEGs described above were enriched in ‘plant–pathogen interaction’, ‘plant hormone signal transduction’, and ‘MAPK signaling pathway—plant’ (Figure 6a). The 356 specific DEGs of R6 were enriched in ‘plant–pathogen interaction’, ‘plant hormone signal transduction’, ‘MAPK signaling pathway—plant’, and ‘benzoxazinoid biosynthesis’ (Figure 6b), while 108 specific DEGs of S8 were not enriched in any pathways. The common DEGs of R6 participated in ‘plant–pathogen interaction’, ‘MAPK signaling pathway—plant’, ‘plant hormone signal transduction’, and ‘amino sugar and nucleotide sugar metabolism’, which were closely related to disease resistance, at multiple timepoints. The common DEGs of S8 participated in the same pathways, except for ‘amino sugar and nucleotide sugar metabolism’ (Figure S8a). To dissect the gene co-expression at each time point, we extracted specific DEGs and investigated the pathway enrichment for each timepoint. Combing the enriched pathways, analyzed at multiple timepoints, those of R6 were enriched regarding ‘plant–pathogen interaction’ and ‘plant hormone signal transduction’ pathways (Figure S8b), while the DEGs of S8 were enriched in ‘plant–pathogen interaction’ and ‘monoterpenoid biosynthesis’ pathways (Figure S8c). These results indicated that DEGs played an essential role in the regulation of hormones or metabolites for disease resistance. To study the difference in resistance, we also investigated KEGG pathway enrichment between two inbred lines. The KEGG pathways of common DEGs from R6 and S8 were enriched at multiple timepoints in ‘metabolic pathways’ and ‘biosynthesis of secondary metabolites’ (Figure S9a). The specific DEGs of R6 and S8 at these timepoints were enriched in ‘metabolic pathways’, and ‘biosynthesis of secondary metabolites’ (Figure S9b,c). These results suggested that the biosynthesis of secondary metabolic pathways was important in the gray leaf spot response, while the DEGs of R6 enriched more pathways than those of S8, including amino acid, starch and sucrose, and hormone biosynthesis.

### 2.6. Co-Expression Network of Genes Related to C. zeina Resistance

**Figure 7:**
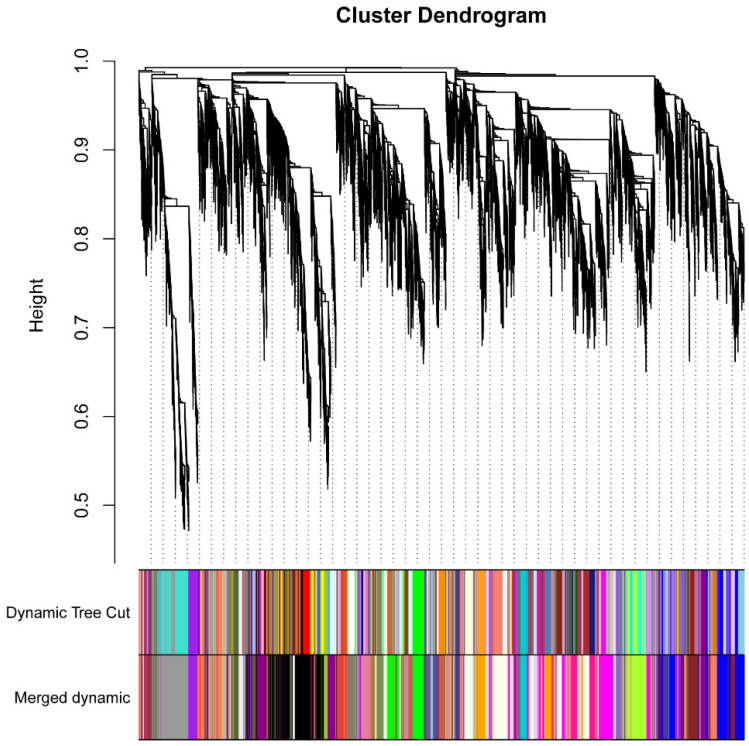
Gene cluster dendrograms and module detection. From the top to the bottom are clustering dendrograms of genes based on the topological overlap, gene modules of dynamic tree cut, gene modules of merged dynamic tree cut, created by merging similar modules.

**Figure 8:**
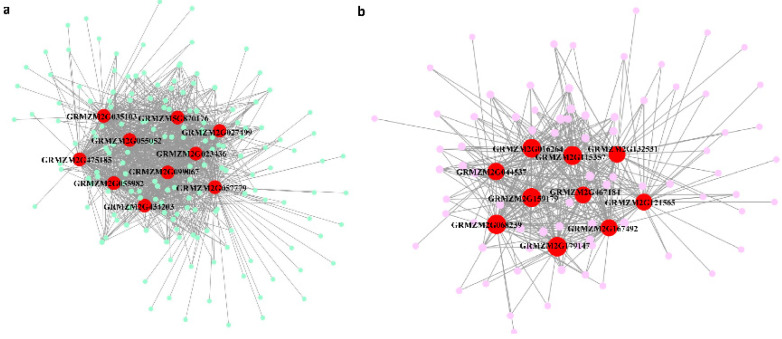
Co-expression network of related gene modules grey60 (**a**) and purple (**b**). Only hub genes from two modules were exhibited in the network.

To investigate the gene regulation related to gray leaf spot resistance, a total of 18,873 highly expressed genes were collected for WGCNA analysis. The sample cluster diagram showed that no outliers were detected in our transcriptomes (Figure S10). To construct the scale-free network, the weighted value was set as 12 for the adjacent matrix, according to the soft threshold of scale-free topology (Figure S11a,b). The dynamic tree cut method was used for gene module division, setting the gene number threshold as 30, and the similar expression pattern was merged by an eigenvalue distance of less than 0.2 (Figure 7). Different color bars represent different gene modules, and the gray bar means the module cannot be merged into other modules. Overall, 45 modules, containing from 41 to 1668 genes, were obtained in our study (Table S3). Among these, the Black module contained the most genes, while the Lightpink2 module contained the fewest genes. Moreover, most modules contained no DEGs, although Grey60 contained 445 DEGs, which was the highest number of DEGs (Table S3). The correlation between modules and traits demonstrated that six modules showed high correlation. The correlation coefficients of Purple and CK1, Thistle1 and S8-240h, Greenyellow and R6-6h, Orange and S8-6h, Magenta and R6-6h, Grey60 and CK2 were higher than 0.80 (Figure S12). To investigate the modules related to disease resistance, GO-term enrichment analysis was performed for the genes in each module. The results indicated 13 modules, including Black, Darkmagenta, Darkolivegreen, Darkolivegreen4, Greenyellow, Grey60, Lightpink2, Magenta, Magenta4, Mediumpurple4, Palevioletred2, Purple, and Yellowgreen, were related to disease resistance. These were the GO terms for BP enrichment in ‘response to abiotic stimulus’, ‘response to stress’, and ‘response to endogenous stimulus’ (Table S4). KEGG pathway enrichment was also analyzed for all the modules. The results showed that an essential pathway, ‘plant–pathogen interaction’, was enriched in Grey60 and Purple modules (Table S5). The hub genes were all DEGs, determined as the top ten genes for connectivity in each module. These hub genes were extracted for the construction of the co-expression network (Figure 8, Table S6). It is worth noting that some of the hub genes showed a close relationship to disease resistance. For example, GRMZM2G027499 was a homolog of *ENHANCED DISEASE RESISTANCE4 (EDR4)* in sorghum and Arabidopsis, which plays an important role in resistance to powdery mildew, was required by salicylic acid signaling [41]. GRMZM2G055052, encoding a RING-type E3 ubiquitin transferase, and its homologous gene, was shown to function in the immune response pathway by targeting BIK1 for degradation [42]. The homologous gene *PBL13* of GRMZM2G057779 was a Serine/Threonine Protein kinase that negatively regulates immune responses in Arabidopsis [43]. GRMZM2G121565 encodes a Putative inactive leucine-rich repeat receptor-like protein kinase; the homolog of this gene modulates brassinosteroid signaling [44], and is required for innate immunity to hemibiotrophic and biotrophic pathogens [45]. GRMZM2G016264 and GRMZM2G159179 both encode putative cytochrome P450 superfamily protein; the homolog CYP94C1 catalyzes the oxidation steps of hormone jasmonoyl-isoleucine and participates in the jasmonate hormonal pathway, which is important for plant defense and development [46]. GRMZM2G167492 encodes putative NAC domain transcription factor NACTF97, contributing to the oxidative stress response, as well as participating in the regulation of plant development and the abiotic stress response [47]. GRMZM2G467184 encodes a putative calcium-binding protein; a similar protein in wheat has been validated to confer resistance to Fusarium head blight [48]. In summary, the predicted function of hub genes demonstrated that hormone signaling, calcium signaling pathways, and transcription factors played a central role in GLS resistance.

### 2.7. Identified Resistance-Related Genes Response to C. zeina from DEGs

**Figure 9:**
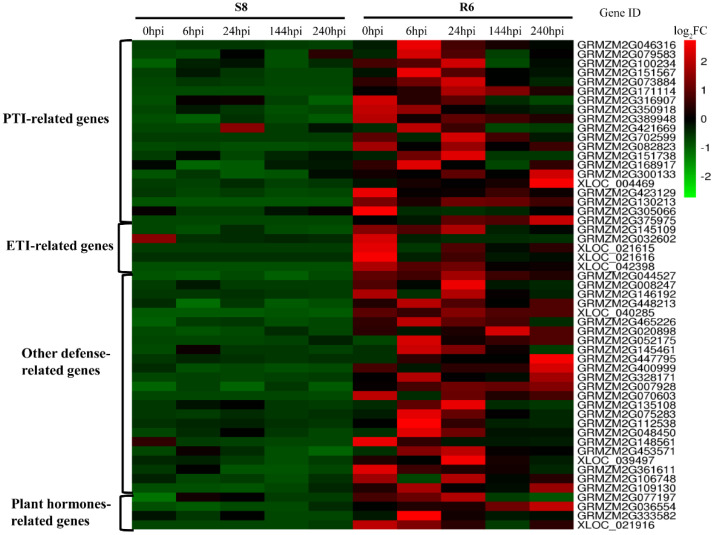
Heat maps of gene transcripts in GLS-sensitive (S8) and GLS-resistant (R6) lines after *C. zeina* infection [*p* < 0.001 and log2FC (fold change) > 1.0 or < −1.0]. The PTI-, ETI-, defense-related, and hormone-related genes whose expression differed significantly are shown. Red indicates higher transcript levels.

To identify disease-resistance-related genes after *C. zeina* infection according to the transcriptional level, a total of fifty-three putative defense response genes were identified between the R6 and S8. The contents of sixteen Leucine-rich repeat (LRR) or similar genes that were close homologs of *flagellin sensing 2 (FLS2)* and *EF-Tu* receptor (EFR), three *MAPK* or *MAPK*-like genes, five ETI-related genes, four plant-hormone-related genes, and twenty-four other defense-related genes that were differentially expressed between R6 and S8 were analyzed (Figure 9, Table S7).

The transcripts of the two RPP genes (XLOC_021615 and XLOC_021616), one RPM1-like gene (XLOC_042398), two LRR or similar genes (GRMZM2G171114 and GRMZM2G702599), MAPK gene (GRMZM2G375975), and MYC2 (XLOC_021916) were specifically expressed in the GLS-resistant line R6 (Figure 9, Table S6). Other defense-related genes, such as the *PEN1* gene (GRMZM2G044527), *TGA* gene, and *PEN2*-like genes (GRMZM2G146192 and GRMZM2G008247) were highly expressed in the GLS-resistant line (R6) (Figure 9, Table S6).

### 2.8. Integration of QTL Mapping and DEGs to Find the Candidate Genes’ Response to C. zeina

In previous QTL-mapping, four GLS-resistance QTLs (*qRgls.CH-1, qRgls.CH-2, qRgls.CH-4, and qRgls.CH-6*) were identified in different chromosomes [49]. Candidate DEGs identified from RNA-seq that were consistently located in the QTL interval were considered consensus genes, which were more reliable for further analysis. Using the physical location of QTL (Table S8), four genes (GRMZM2G702599, GRMZM2G447795, GRMZM2G171114, and GRMZM2G135108) from fifty-three putative defense genes and one gene (GRMZM2G044537) from twenty DEG hub genes (Table S6, S7 and S9) were mapped within the previous QTL regions. Interestingly, GRMZM2G702599 and GRMZM2G171114 were specifically expressed in R6 (Figure 9, Table S7), and were both putative Leucine-rich repeat protein kinase family proteins, which are highly likely to be resist to GLS. Moreover, another three genes located in QTL regions, i.e., GRMZM2G044537, which was putative RING zinc finger protein-like, GRMZM2G447795, which was putative Xylanase inhibitor protein 1, and GRMZM2G135108, which was putative Peroxidase, are worthy of note as well.

## 3. Discussion

Since the maize defense mechanism against *C. zeina* is far too unraveled to date, we first compared the physiological characterization of key enzymes related to plant defence. According to the intensity of response, timepoints were chosen to compare the specific transcriptome profiling of two inbred maize lines that differed in GLS resistance after *C. zeina* infection. After QTL mapping and WGCNA analysis, many candidate genes were identified, which provided important information regarding their mechanism of resistance against GLS.

In our study, the PAL, CAT and POD of the resistant line R6 showed higher activity than the susceptible line S8. Although both the resistant and susceptible lines showed an intense response after *C. zeina* infection at different timepoints, the resistant line, R6, was more dramatically changed compared with the susceptible line, S8, except for FP content. These results suggest that the different resistance levels of R6 and S8 may be related to two physiological mechanisms. One is the initial activity or content: the resistant lines, R6, always showed higher activity or content than the susceptible line S8; the higher activity or content of enzymes led to a superior defense when *C. zeina* invaded maize leaves. The other is the intensity of activity changes after *C. zeina* infection. Higher intensity implies a stronger defense, with a higher accumulation of phenylpropanoid pathway products, and stronger hydrogen peroxide degradation. The PAL, CAT, and POD might be applicable physiological signs when screening the response of resistant inbred lines to GLS when infected by *C. zeina*.

According to the PAL, CAT, POD, and FP response to *C. zeina* infection in maize leaves, five stages (0, 6, 24, 144, and 240 h) were chosen to investigate the transcriptional changes that occurred in response to *C. zeina* infection in more detail. The GO term showed that the DEGs were significantly enriched in defense responses such as ‘response to biotic stimulus’ and ‘response to stress’, and the KEGG pathway was correspondingly enriched in its ‘plant–pathogen interaction’, ‘MAPK signaling pathway–plant’, and ‘plant hormone signal transduction’, which indicated that the MAPK signaling pathway and hormone signal transduction were essential after *C. zeina* infection. Some studies have shown that MAPK cascades play pivotal roles in plant defense against pathogen attack and are the earliest signaling events after sensing the pathogen [50]. The plant hormone crosstalk can potentially enhance pathogen resistance and overall plant fitness [51]. It is notable that ‘Benzoxazinoid biosynthesis’ is a specific pathway enriched in R6. The pathway participates in the regulation of innate immunity against aphids and fungi in maize [52], which may contribute to the superior resistance of R6.

The specific interactions between plant and pathogen can be addressed by identifying the transcription alterations in critical genes involved in PTI and ETI, such as pathogen-associated molecular pattern (PAMP) receptors (*PRRs*), ETI receptors, salicylic acid (SA)-, jasmonic acid (JA)-, and ethylene (ET)-response genes [25,53,54,55,56]. Two *EFR-* and *FLS2*-like genes, as the putative PAMP receptors, were expressed only in R6. Importantly, the key PTI genes triggered by PAMPs, such as *MAPK* genes, were highly expressed in the GLS-resistant line at all timepoints, and one *MAPK* gene (GRMZM2G375975) was specifically expressed in R6 (Figure 9; Table S6). Genes expressed in the early-stage responses, such as 0 and 6 h, might be more important for GLS resistance than those expressed at the later stages (i.e., 24, 144, and 240 h). In this study, the transcripts of the two *RPP* genes (XLOC_021615 and XLOC_021616) were specifically expressed in the GLS-resistant line. It was validated that the *RPP* genes trigger localized cell death in Arabidopsis upon recognition of downy mildew avirulence genes [57]. In Arabidopsis, *RPM1* encodes an intracellular immune sensor that is induced in response to *Pseudomonas syringae*, and attack by this pathogen leads to the expression of *RPM1* disease resistance genes [58,59]. In the present study, one *RPM1*-like gene (XLOC_042398) was specifically expressed in the GLS-resistant line R6. *PENETRATION 1 (PEN1)*, *PEN2*, and *PEN3* have been demonstrated to play an essential role in non-host resistance [54]. One *PEN1* gene (GRMZM2G044527), two *PEN2*-like genes (GRMZM2G146192 and GRMZM2G008247) were up-regulated in the GLS-resistant line at all timepoints (Figure 9, Table S6). WRKY TFs have also been shown to be involved in plant immune responses to bacterial pathogens [60]. The *LOX1*-mediated pathways are crucial for lipid peroxidation during plant defense responses to pathogen infection [61]. The transcript levels of two *LOX1* (GRMZM2G106748 and GRMZM2G109130) genes were low at all sample timepoints in the GLS-susceptible line S8 (Figure 9, Table S6). In contrast, these defense-related genes were highly expressed in the GLS-resistant line (R6). JA/ET primarily participates in the deterrence of herbivores and resistance to necrotrophic pathogens, whereas SA is primarily involved in resistance to biotrophic and hemibiotrophic pathogens [62]. The expression of a maize non-expressor of the PR1 gene [*NPR1*-like gene (GRMZM2G077197)] was higher in terms of resistance than the sensitive line at 0, 6, and 24 h (Figure 9, Table S6); *NPR1* is an important regulatory component in the SA signaling. The core JA-signaling component *MYC2* had a significantly higher resistance than the sensitive line all the time; hence, upregulation of *MYC2* related to JA-signaling was shown in the GLS-resistant line. One reason for the elevated expression of JA signaling components may be that the leaf scrubbing before spraying *C. zeina* spore suspension was perceived as wounding. Alternatively, endogenous levels of JA may be elevated in the GLS-resistant line compared with the GLS-susceptible line.

GLS is a severe disease, resulting in an extreme reduction in the grain yield of maize. In our study, the transcriptomic atlases at different timepoints were analyzed for the DEGs identified after *C. zeina* infection. Combining QTL mapping and WGCNA, we analyzed candidate genes, the homolog of which was related to immune response and disease resistance. Other than the single functional genes, transcription factors such as MYBs, and hormone-related genes, such as JA and ethylene-related genes, were involved in GLS response and resistance. These results indicated the complex defense mechanisms against GLS and provided gene resources for further validation.

## 4. Materials and Methods

### 4.1. Plant Materials and Disease Resistance Scoring

The inbred maize lines 646 (resistant line, named R6) and 08-641 (susceptible line, named S8) were used in the current study. R6 was developed at WUGU Seed Company Limited with stable GLS resistance, whereas S8 is a GLS-susceptible inbred line developed at the Maize Research Institute of Sichuan Agricultural University. The GLS resistance of the two maize inbred lines was evaluated across Luding and Baoshan (Sichuan and Yunnan Province, China) in 2012 and 2013, respectively. Three replicates were set for each genotype, with more than 200 plants per replicate. All the plants were scored for GLS with 10-day intervals, starting 15 days after silking. The GLS rating ranged from 1 to 9, with 1 and 3 being considered resistant, 5, 7, and 9 representing susceptibility [63].

### 4.2. The C. zeina Strain Preparation and Inoculation

*C. zeina,* used as inoculum, was collected from fresh leaves infected by *C. zeina* in Luding, Sichuan Province, an area with natural and heavy gray leaf spot infection. The fungal spores were collected by washing leaves infected by *C. zeina*, and the suspension was concentrated. The spore suspensions used for inoculation were adjusted to a density of 200 × 104 spores per mL using a hemocytometer.

All sample plants were scrubbed with emery (600 mesh) and then inoculated at the flowering stage by spraying a suspension of spores (approximately 3–5 mL per plant, on 3 consecutive days) on the leaf surface of each plant; the relative humidity of the growth chamber and temperature were adjusted to 100% and 25 °C (high humidity and mild temperature) to ensure the occurrence of GLS.

### 4.3. Antioxidant Enzymes Activity and Proline Assay

After inoculation, maize leaves of each genotype were collected at 0 (control: the spore suspension sprayed and then sampled immediately), 1, 2, 4, 6, 8, 10, 12, 24, 48, 96, 144, 192, 240, 288 h post-inoculation. All the samples were immediately frozen in liquid nitrogen and stored at −80 °C.

Leaf materials with fresh weights of 1g were homogenized in phosphate saline buffer (pH 7.8, 0.05 M) containing β-mercaptoethanol (2 μL) and a trace of polyvinylpyrrolidone (PVP). The homogenate was filtered through cheesecloth and centrifuged at 12,000× *g* at −4 °C for 10 min, and the supernatant was collected for enzyme activity assay.

The PAL activity assay used the method described by Wang et al. [64]. One unit of the enzyme was defined as an increase in absorbance of one unit per min. The enzyme activity was expressed as units per mg of soluble protein.

The CAT activity was assayed by measuring the initial rate of H_2_O_2_ disappearance using the method of Beers and Sizer [65]. The catalase assay reaction mixture contained 0.05 mM sodium phosphate buffer (pH 7.0), 20 μL enzyme extract/mL and 1 mM H_2_O_2_. Degradation of H_2_O_2_ was followed by measuring a decrease in absorbance at 240 nm, and the activity [U (mg protein) ^−1^] was calculated using a molar absorption coefficient of 40 mM^−1^ cm^−1^ for H_2_O_2_.

The peroxidase (POD) activity was determined using the method of Upadhyaya et al. [66] in a 3.9 mL reaction mixture containing 50 mM phosphate buffer (pH 7.0), 28 mL of guaiacol, 100 μL enzyme extract, and 19 μL H_2_O_2_. The absorbance was monitored at 420 nm for at least 2 min at 30 s intervals; absorbance changes of 0.01 represented one unit of POD activity.

The FP was analyzed according to the method of Bates et al. [67]. The chromophore containing the FP was aspirated and added to a test tube, then warmed to room temperature, and the absorbance was measured at 520 nm on a spectrophotometer (Hitachi U-2001, Japan). A standard curve was constructed by running the proline standards. All assays were conducted for three biological replicates for each treatment.

### 4.4. Samples Collection and RNA Isolation

Based on the antioxidant enzyme activity assay results, the leaf samples were collected at 0, 6, 24, 144, and 240 h from inoculated and control plants to obtain the enriched expression library. Total RNA was extracted using Trizol reagent (Invitrogen, Carlsbad, CA, USA) according to the manufacturer’s instructions, and mRNA was enriched by Oligo (dT) beads. The enriched mRNA was fragmented and reverse-transcribed into cDNA with random primers. Second-strand cDNAs were synthesized by DNA polymerase I, RNase H, dNTP, and buffer. The cDNA fragments were purified using a QiaQuick PCR extraction kit, end-repaired, poly (A)-supplemented, and ligated to Illumina sequencing adapters. Two independent biological replicates were used for RNA-seq experiments.

### 4.5. RNA Sequencing and Data Analysis

RNA library sequencing was performed using an Illumina HiSeqTM 2500 by Gene Denovo Biotechnology Co. (Guangzhou, China). Raw reads were filtered by removing reads with adaptor sequences, reads in which the percentage of unknown bases (N) was greater than 10%, and low-quality reads. Clean reads were used for mapping, calculation, and normalization of gene expression. All the clean reads in each sample were mapped to B73 (RefGen_V3) genomic DNA sequence using Tophat2 (2.1.1) [68]. Gene abundances were quantified by software RSEM (v1.3.1) [69]. edgeR package (version 3.22.5) (http://www.r-project.org/, accessed on 15 February 2021) was used to identify differentially expressed genes among the samples [70]. We identified genes with a fold change ≥ 2 and a false discovery rate (FDR) < 0.05 in a comparison as significant DEGs. All DEGs were mapped to GO terms in the Gene Ontology database (http://www.geneontology.org/, accessed on 15 February 2021); the gene numbers were calculated for every term, and significantly enriched GO terms in DEGs, in comparison with the genome background, were defined by hypergeometric test. The calculated *p* values were subjected to FDR correction, taking FDR ≤ 0.05 as a threshold.

### 4.6. Validation of RNA-Seq Results by qRT-PCR

Quantitative real-time PCR was used to validate the DEGs between the resistant and susceptible genotypes. RNA extraction and first-strand cDNAs synthesis were carried out as described above. *Actin1* (GRMZM2G126010) was used as the endogenous control. The corresponding primers were designed using Primer 5 software and are listed in Table S10. Each 16 μL PCR mixture contained 8 μL of 2 × real-time SYBR Green I PCR Mix, 0.2 μL of each primer, and an appropriate quantity of cDNA. The amplification programs were set according to the standard ABI ViiA™7 system protocol: 95 °C for 2min; followed by 40 cycles of 94 °C for 10 s, 60 °C for 10 s and 72 °C for 40 s. The threshold cycles (Ct) of each tested gene were averaged for triplicate reactions, and the values were normalized according to the Ct of the control products of the Actin1 gene. The statistical analysis was performed using the 2^−ΔΔCT^ method [71].

### 4.7. WGCNA Analysis of RNA-Seq Data from Multiple Timepoints

The RNA-Seq data including two biological repeats were collected for WGCNA analysis. The genes with low FPKM (<5) at all timepoints were filtered because they were not meaningful for downstream analysis. The correlation coefficient between each pair of genes was calculated by the formula: Smn = cor(xm, xn), S = [Smn]; the Smn represented the Pearson correlation coefficient between gene m and gene n, while S represented similar matrix. WGCNA R package (version 1.6.9) was used for WGCNA analysis [72]. To make the network conform to the scale-free network distribution, the suitable weighted value was estimated by the function ‘pickSoftThreshold’ of WGCNA according to a threshold of 0.8. The topological overlap matrix (TOM) was transformed from the adjacent matrix. Inverse matrix was calculated by TOM, and use ‘hclust’ to perform hierarchical clustering. The cluster tree was cut by Dynamic Tree Cut, and the gene cutoff in each module was set to 30. The modules with a similar expression pattern (>0.8) were merged as the final coexpression module. GO term and KEGG pathway enrichment were performed for each module, and modules were extracted for the hub gene screen according to the result. The hub genes were screened as the common DEGs from the top ten genes in terms of connectivity in the related modules. The coexpression network of hub genes was constructed by Cytoscape 3.8.1 [73].

## 5. Conclusions

By using RNA-Seq to characterize the differential expression of genes over five disease stages caused by *C. zeina*, a genome-wide transcription profile of disease development in maize was obtained. Combined with the QTL, five genes were identified as candidate genes for GLS resistance. The molecular functions of these genes and their associated pathways provided insights into the molecular mechanisms of the GLS development in maize in response to *C. zeina* infection. These results will facilitate future analyses of the resistance mechanism and will contribute to breeding maize that is resistant to gray leaf spot.

## Data Availability

The datasets were deposited in the National Center for Biotechnology Information (NCBI) Sequence Read Archive (SRA) under accession number SRA: SRP111105 (https://www.ncbi.nlm.nih.gov/sra/?term=SRP111105, accessed on 15 September 2021).

## Supplementary Materials

The following are available online at https://www.mdpi.com/article/10.3390/plants10112257/s1, Figure S1: Correlation tests for the replicates. The x and y axis feature FPKM in each replicate. R2 is the square of Pearson’s correlation coefficient. Note: CK = 0 h; 6h = 6 h; 1d = 24 h; 6d = 144 h; 10d = 240 h. Figure S2: Volcano plot of DEGs between maize lines R6 and S8 at various times after inoculation with *C. zeina*. The Log2FC (fold change) in red represents significantly upregulated and green shows significantly downregulated transcripts (FDR < 0.05). Figure S3: Verification of RNA-seq results by qRT-PCR. The Y axis (left) indicates the results of qRT-PCR, and the Y axis (right) indicates the results of transcriptome; the X-axis indicates the sample time, CK: control, 6 hpi: 6 h post-inoculation, 24 hpi: 24 h post-inoculation. S8: GLS-sensitive line, R6: GLS-resistant line. Figure S4: GO term enrichment of DEGs from R6 at different timepoints. (a–d) GO term enrichment of DEGs from 6 h, 24 h, 144 h, and 240 h. Figure S5: GO term enrichment of DEGs from S8 at different timepoints. (a–c) GO term enrichment of DEGs from 6 h, 24 h, and 240 h. Figure S6: GO term enrichment of specific DEGs from R6 at each timepoint. (a–d) GO term enrichment of specific DEGs from 6 h, 24 h, 144 h, and 240 h. Figure S7: GO term enrichment of specific DEGs from S8 at each timepoint. (a–d) GO term enrichment of specific DEGs from 6 h, 24 h, 144 h, and 240 h. Figure S8: KEGG pathway enrichment of DEGs from R6 and S8 at different timepoints. (a) KEGG pathway enrichment of common DEGs from multiple timepoints from R6 and S8. (b) KEGG pathway enrichment of DEGs from R6 at different timepoints. (c) KEGG pathway enrichment of DEGs from S8 at different timepoints. Figure S9: KEGG pathway enrichment of common and specific DEGs from R6 and S8 at different timepoints. (a) KEGG pathway enrichment of common DEGs identified from R6 and S8 at each timepoint. (b) KEGG pathway enrichment of specific DEGs of R6 at each timepoint compared with S8. (c) KEGG pathway enrichment of specific DEGs of S8 at each timepoint compared with R6. Figure S10: The sample clustering tree of samples collected for WGCNA. Figure S11: Determination of soft threshold for scale-free network. (a) The number scatter plot between soft threshold and the index of scale-free network model. (b) The number scatter plot between soft threshold and mean connectivity. Figure S12: The correlation relationship heatmap between module eigengenes and expression trait at different timepoints. In each brick, the upper numbers are correlation coefficients; lower numbers are *p* values. Table S1: The reads statistics, mapping and expression genes’ summary for R6 and S8 after *C. zeina* inoculation. Table S2: Gene names of stable common DEGs in R6 at all timepoints, stable common DEGs in S8 at all timepoints, and overlapped common DEGs at all timepoints between R6 and S8. Table S3: Gene information for each module. Table S4: GO term enrichment of genes across all modules. Table S5: KEGG pathway enrichment of genes across all modules. Table S6: Hub genes from gene modules grey60 and purple. Table S7: Putative defense response genes that showed significant differences in expression between the GLS-susceptible and GLS-resistant lines after *C. zeina* infection. Table S8: QTL information for GLS resistance. Table S9: A list of consistent genes located within QTL for GLS. Table S10: List of genes whose transcription profiles were evaluated by RT-qPCR. For each gene, the forward (fw) and reverse (rev) primer sequences are provided.
