# Supplementary material for: Transcriptomic Analysis Reveals Candidate Genes Responding Maize Gray Leaf Spot Caused by Cercospora zeina
Source: Plants (Basel). 2021 Oct 22;10(11):2257. doi: 10.3390/plants10112257 (PMC8625984; doi:10.3390/plants10112257)
Supplement: Supplementary file 1 [file plants-10-02257-s001.zip › Table S1.pdf]

Inbred lines	Time points	Replica tions	Total Reads	Mapped Reads	Unique Mapped Reads	Mapping Rates (%)	Unique Mapping Rates (%)	Number of expression genes
**R6**	0 hpi	repl_1	16147756	11160006	10808262	69	67	25264
		repl_2	31961426	22677066	22067928	71	69	26435
	6 hpi	repl_1	25996212	18787010	18426252	72	71	26670
		repl_2	19875490	13819887	13572709	70	68	25778
	24 hpi	repl_1	27744036	18450741	17955011	67	65	25864
		repl_2	28210470	20063678	19594558	71	69	25754
	144 hpi	repl_1	21671620	15103986	14748580	70	68	26105
		repl_2	19969828	14039598	13647962	70	68	25595
	240 hpi	repl_1	23097500	15812601	15458365	68	67	25724
		repl_2	22919920	14968590	14662914	65	64	26016
**S8**	0 hpi	repl_1	32098076	22735773	21990399	71	69	26822
		repl_2	32233060	22810764	22060430	71	68	26668
	6 hpi	repl_1	28409020	19960031	19417027	70	68	27065
		repl_2	20590866	14681383	14313683	71	70	26633
	24 hpi	repl_1	33311232	23179468	22446594	70	67	26581
		repl_2	33042598	22875556	22211380	69	67	26782
	144 hpi	repl_1	38186826	25908587	25317497	68	66	27529
		repl_2	28872340	20397267	19864117	71	69	27124
	240 hpi	repl_1	31198486	20602020	20158080	66	65	27130
		repl_2	34753676	23383228	22796674	67	66	27187
