# Supplementary material for: Transcriptomic Analysis Reveals Candidate Genes Responding Maize Gray Leaf Spot Caused by Cercospora zeina
Source: Plants (Basel). 2021 Oct 22;10(11):2257. doi: 10.3390/plants10112257 (PMC8625984; doi:10.3390/plants10112257)
Supplement: Supplementary file 1 [file plants-10-02257-s001.zip › Table S10.pdf]

| Gene ID | 5'-3' primer sequences |
|---|---|
| GRMZM2G151567-fw | GTTGTCGGCCAGATACCAGC |
| GRMZM2G151567-rev | TTCCCGATGAATGAAGGCAC |
| GRMZM2G465226-fw | ACTGCCAGCTGATCCACTCC |
| GRMZM2G465226-rev | ACTGCTTCTCGGACACCCAG |
| GRMZM2G145461-fw | GAAGAAGTACTACGGCCGCG |
| GRMZM2G145461-rev | GGCGTCATCCAGAACCAGAC |
| GRMZM2G007928-fw | GACCAGTGCGGCATTTGCAC |
| GRMZM2G007928-rev | AAGAGGCCGTCGGAGATGGA |
| GRMZM2G075283-fw | GCATGTGGAAGGTGGCCTTC |
| GRMZM2G075283-rev | CTTCATGGTGGTGACGCTGC |
| GRMZM2G148561-fw | TGCTGGCTTCCTCGCTCTCC |
| GRMZM2G148561-rev | TGCGGTCCAGCAGGTTGATG |
| GRMZM2G361611-fw | GACATCCTCGCCTGCATGAA |
| GRMZM2G361611-rev | GGCAGGATCTGAGGAGGTCG |
