# Supplementary material for: Transcriptomic Analysis Reveals Candidate Genes Responding Maize Gray Leaf Spot Caused by Cercospora zeina
Source: Plants (Basel). 2021 Oct 22;10(11):2257. doi: 10.3390/plants10112257 (PMC8625984; doi:10.3390/plants10112257)
Supplement: Supplementary file 1 [file plants-10-02257-s001.zip › Table S8.pdf]

QTLs	Chromosomes	Bin	SSR markers	Physical interval
qRgls. CH-1	1	1.05-1.07	Umc2232-umc2237	164,508,927-224,970,667
qRgls. CH-2	2	2.00-2.02	Umc1419-umc1265	945,637-8,019,086
qRgls. CH-3	3	3.04-3.05	Umc1539-umc2266	168,492,776-178,184,934
qRgls. CH-4	4	4.05-4.06	Umc1329-umc1346	135,229,161-166,915,798
qRgls. CH-6	6	6.02-6.05	Bnlg2191-umc2055	91,124,061-132,980,851
