# Supplementary material for: Transcriptomic Analysis Reveals Candidate Genes Responding Maize Gray Leaf Spot Caused by Cercospora zeina
Source: Plants (Basel). 2021 Oct 22;10(11):2257. doi: 10.3390/plants10112257 (PMC8625984; doi:10.3390/plants10112257)
Supplement: Supplementary file 1 [file plants-10-02257-s001.zip › Table S9.pdf]

Genes	Chromosome	Putative protein	position
GRMZM2G702599	4	leucine-rich repeat receptor-like protein kinase family protein	139,022,453:139,023,167
GRMZM2G044537	4	RING zinc finger protein-like	155,937,505:155,938,986
GRMZM2G447795	6	Xylanase inhibitor protein 1	129,268,963:129,270,180
GRMZM2G171114	6	Leucine-rich repeat protein kinase family protein	103,870,389:103,873,917
GRMZM2G135108	6	Peroxidase	125,194,486:125,196,087
