# Supplementary figures and images for: Transcriptomic Analysis Reveals Candidate Genes Responding Maize Gray Leaf Spot Caused by Cercospora zeina
Source: Plants (Basel). 2021 Oct 22;10(11):2257. doi: 10.3390/plants10112257 (PMC8625984; doi:10.3390/plants10112257)

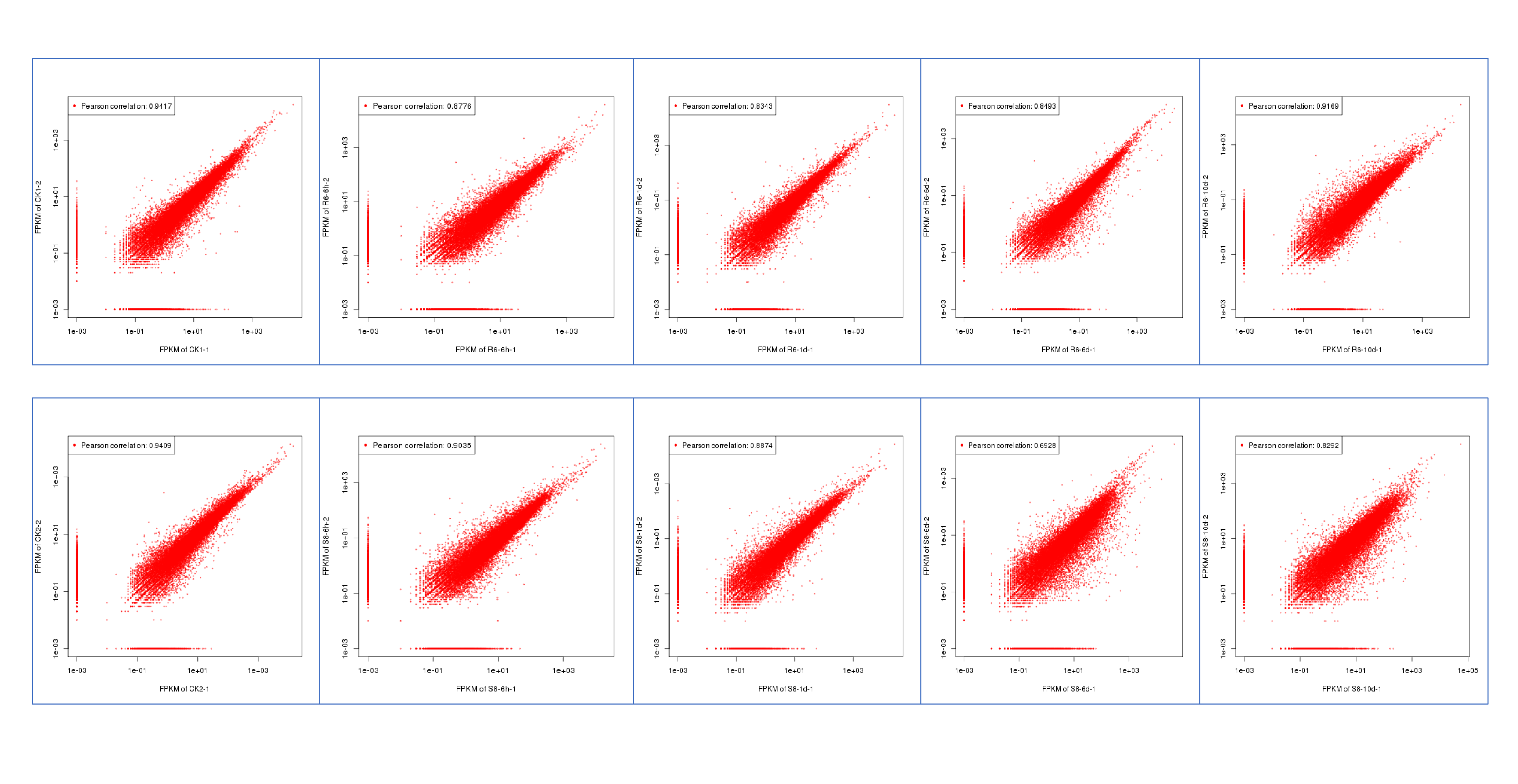

Supplement: Supplementary file 1 [file plants-10-02257-s001.zip › Figure S1.tif]

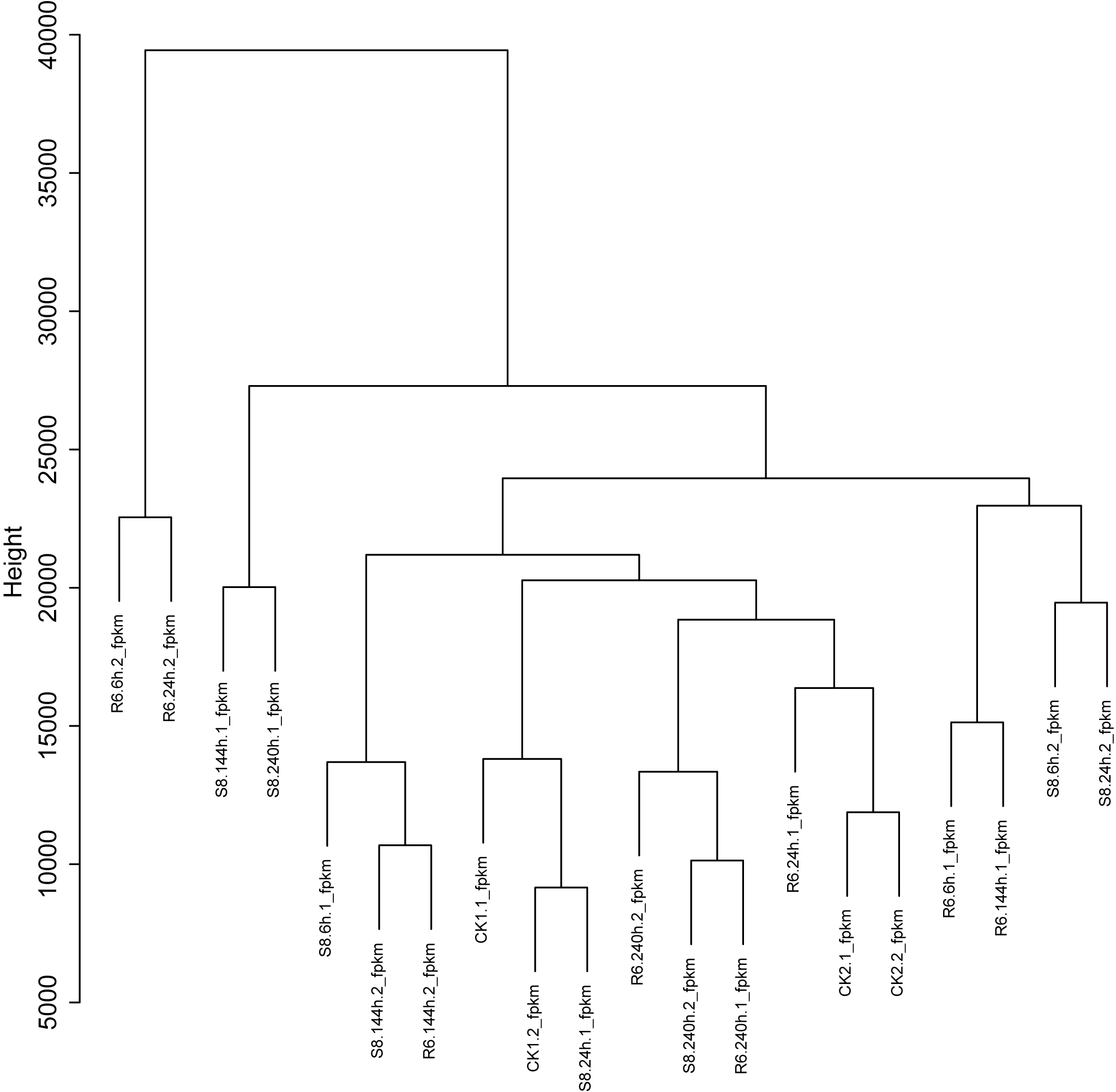

Supplement: Supplementary file 1 [file plants-10-02257-s001.zip › Figure S10.tif]

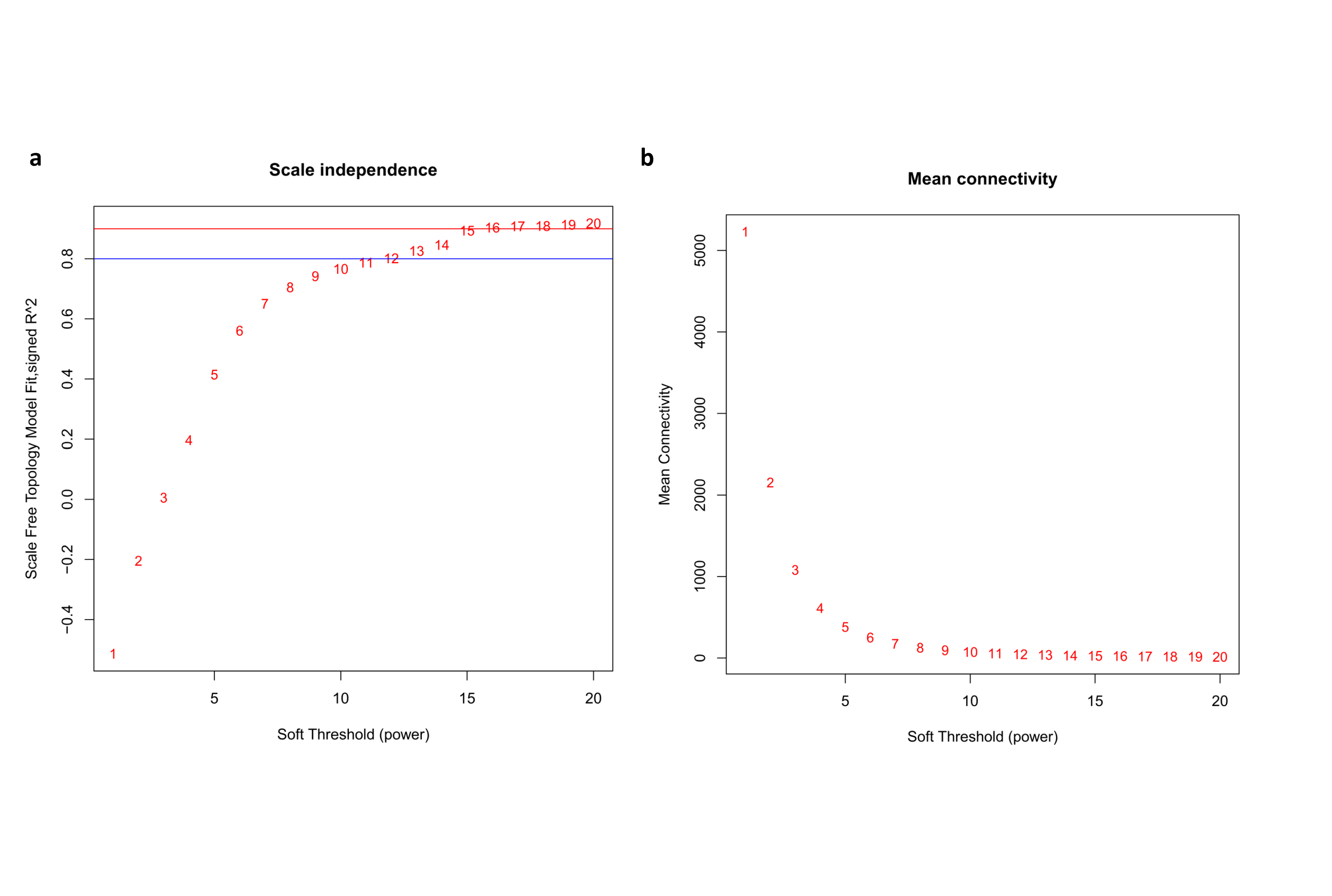

Supplement: Supplementary file 1 [file plants-10-02257-s001.zip › Figure S11.tif]

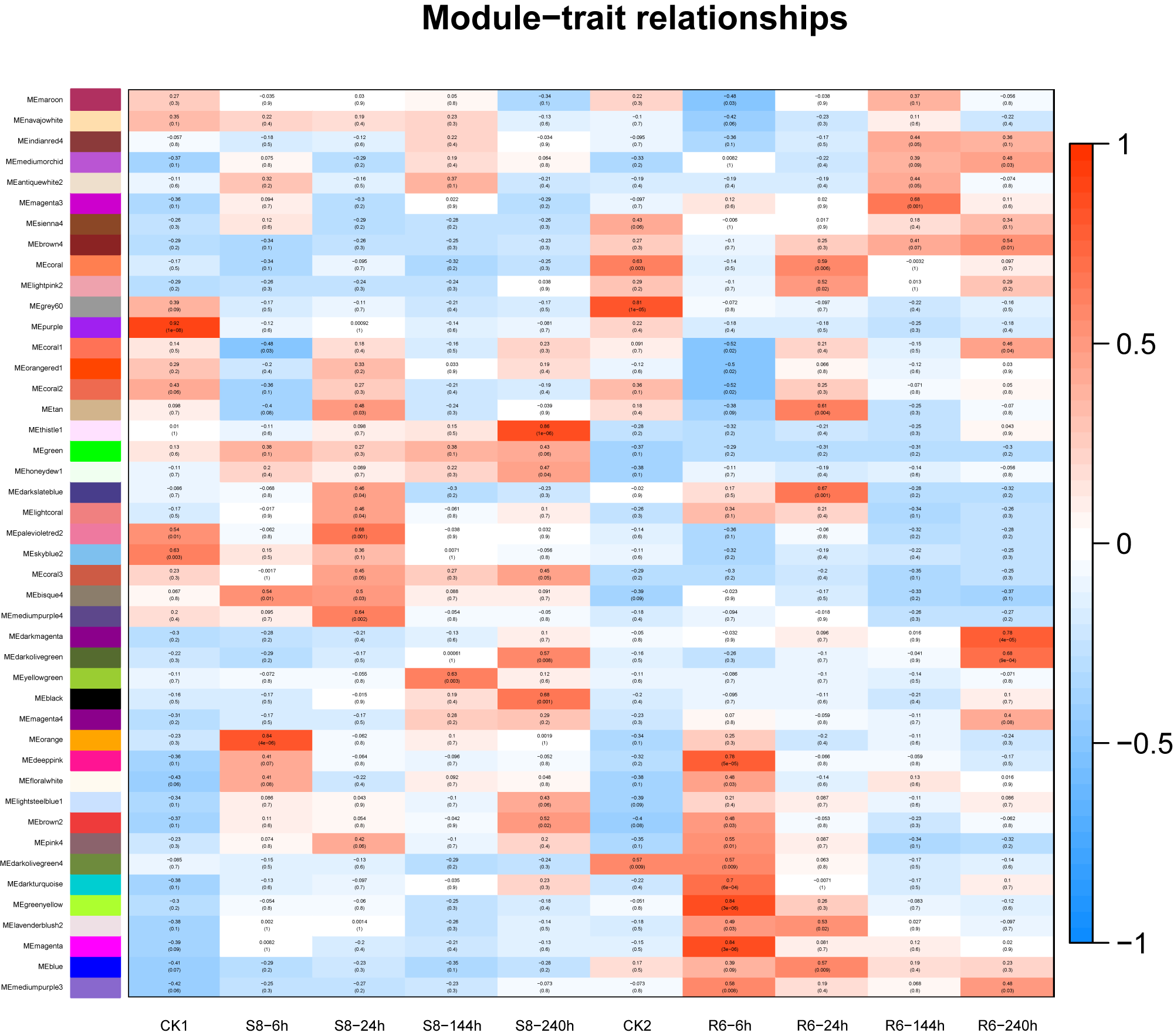

Supplement: Supplementary file 1 [file plants-10-02257-s001.zip › Figure S12.tif]

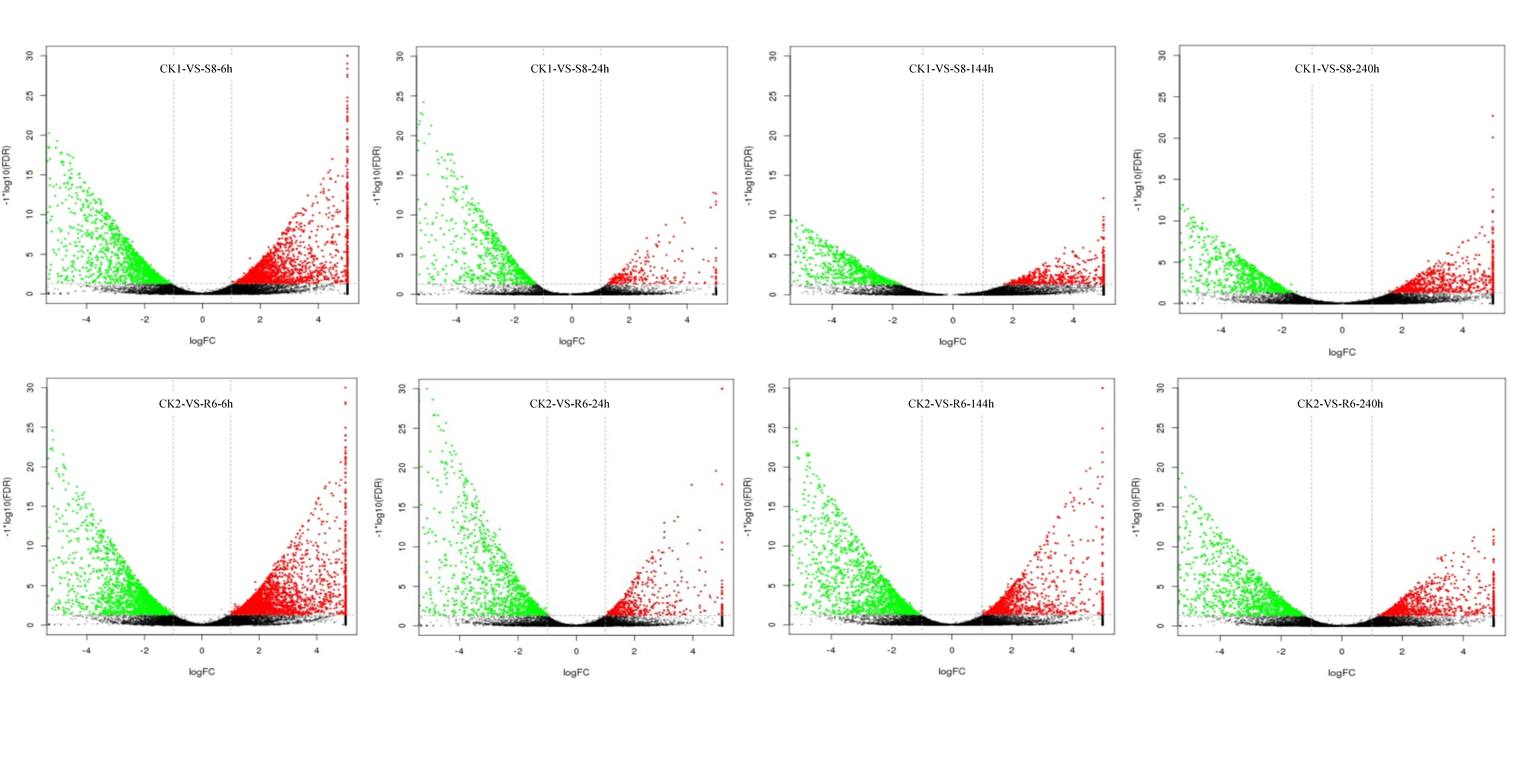

Supplement: Supplementary file 1 [file plants-10-02257-s001.zip › Figure S2.tif]

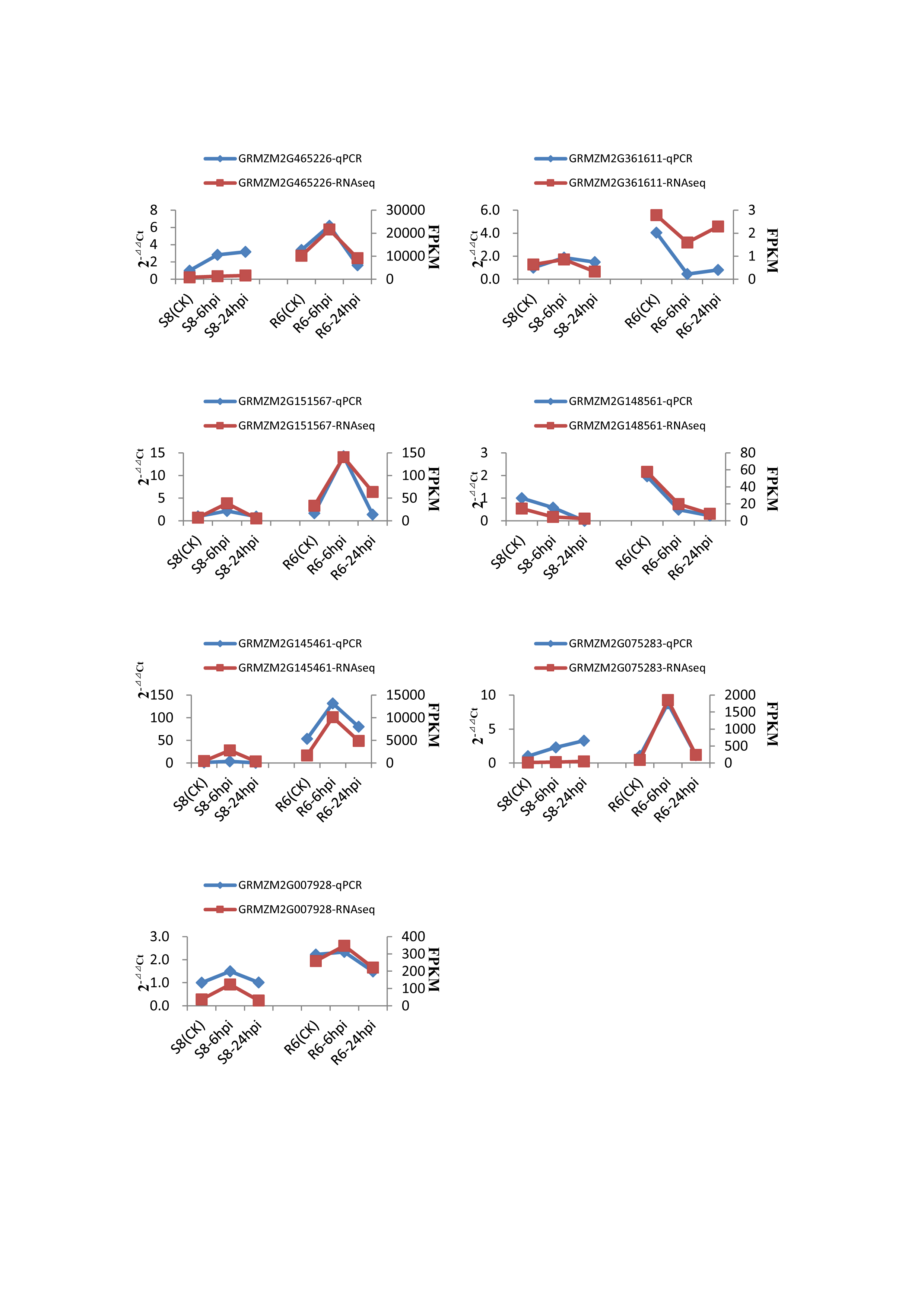

Supplement: Supplementary file 1 [file plants-10-02257-s001.zip › Figure S3.tif]

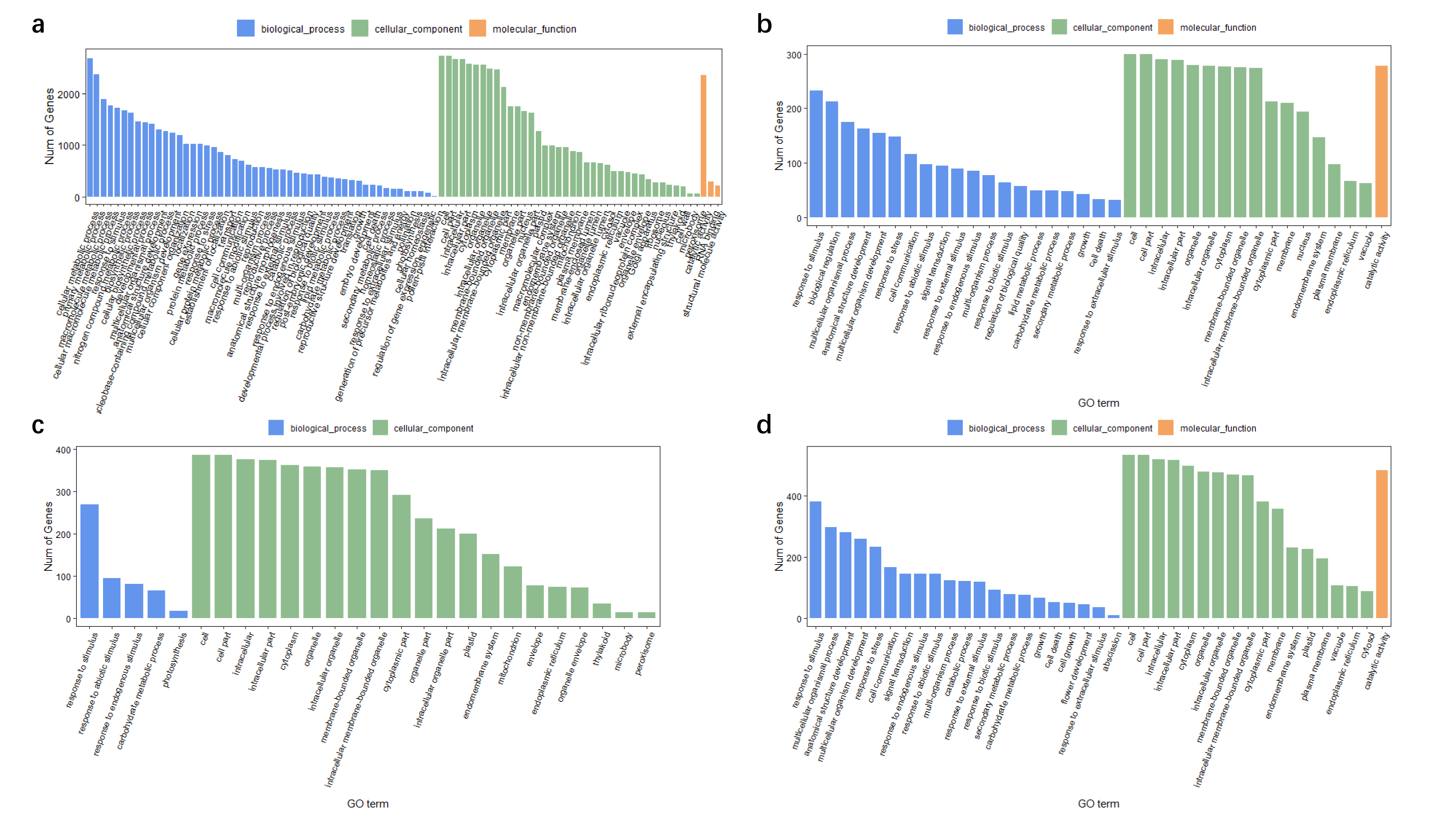

Supplement: Supplementary file 1 [file plants-10-02257-s001.zip › Figure S4.tif]

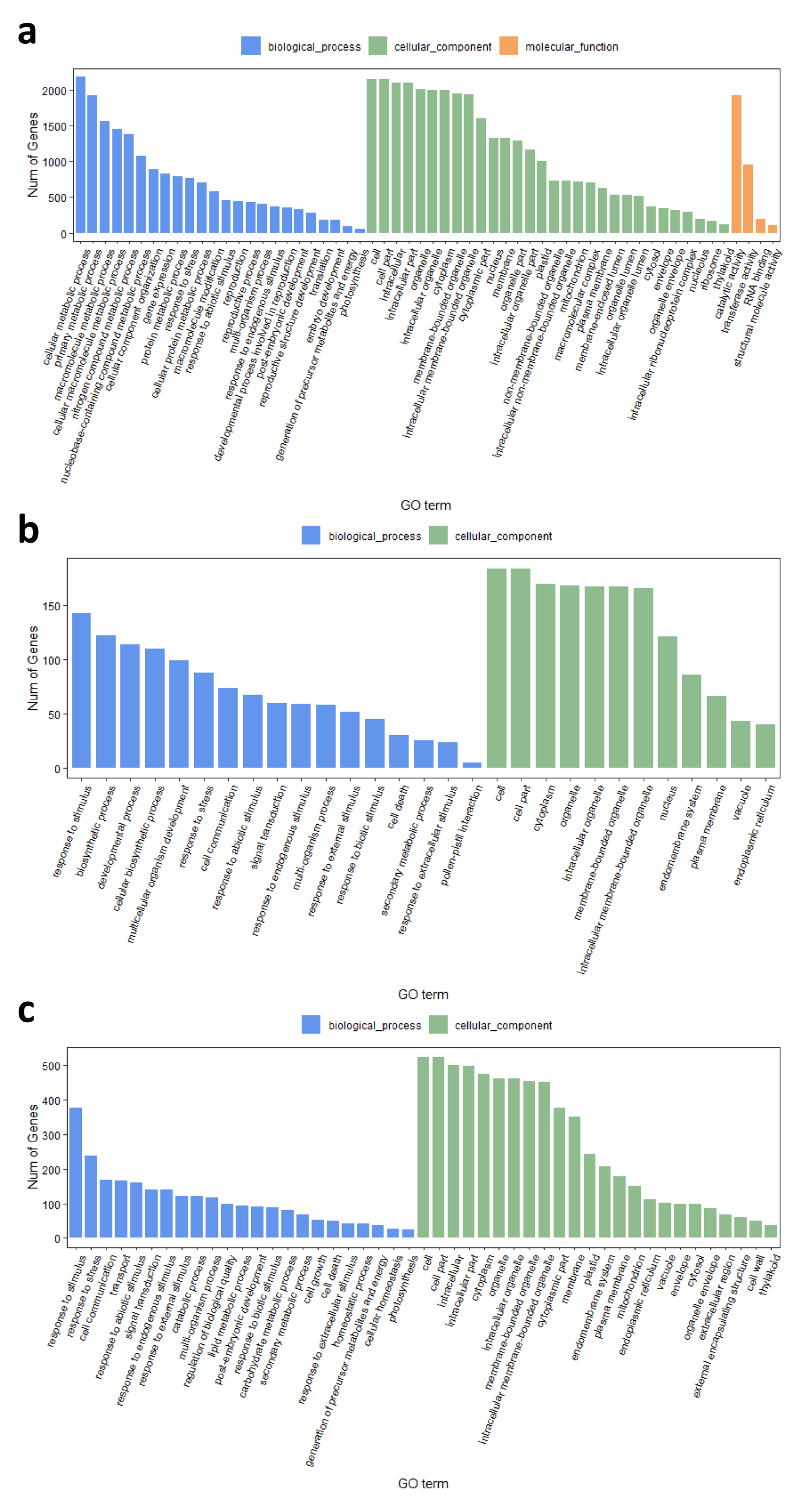

Supplement: Supplementary file 1 [file plants-10-02257-s001.zip › Figure S5.tif]

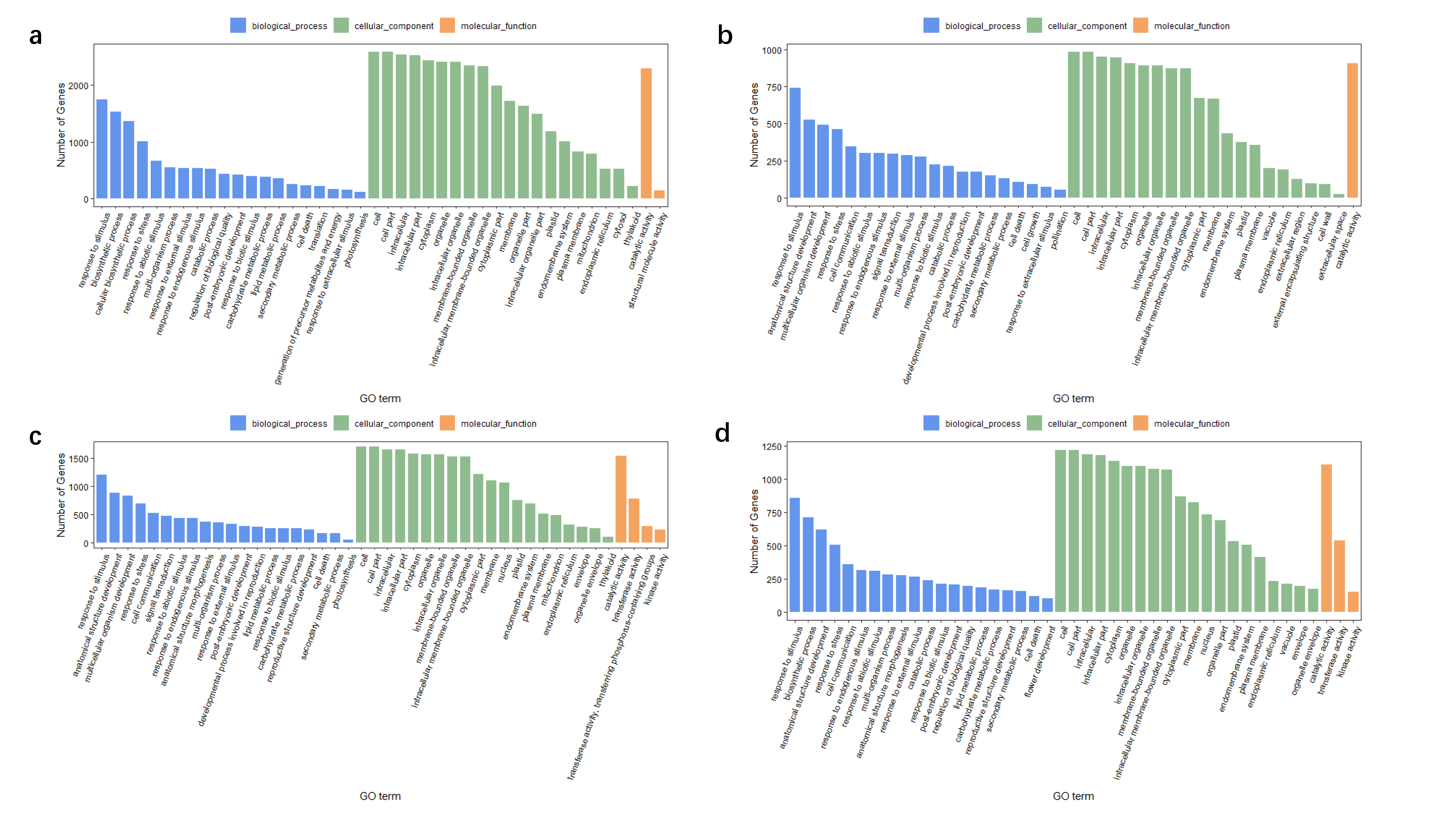

Supplement: Supplementary file 1 [file plants-10-02257-s001.zip › Figure S6.tif]

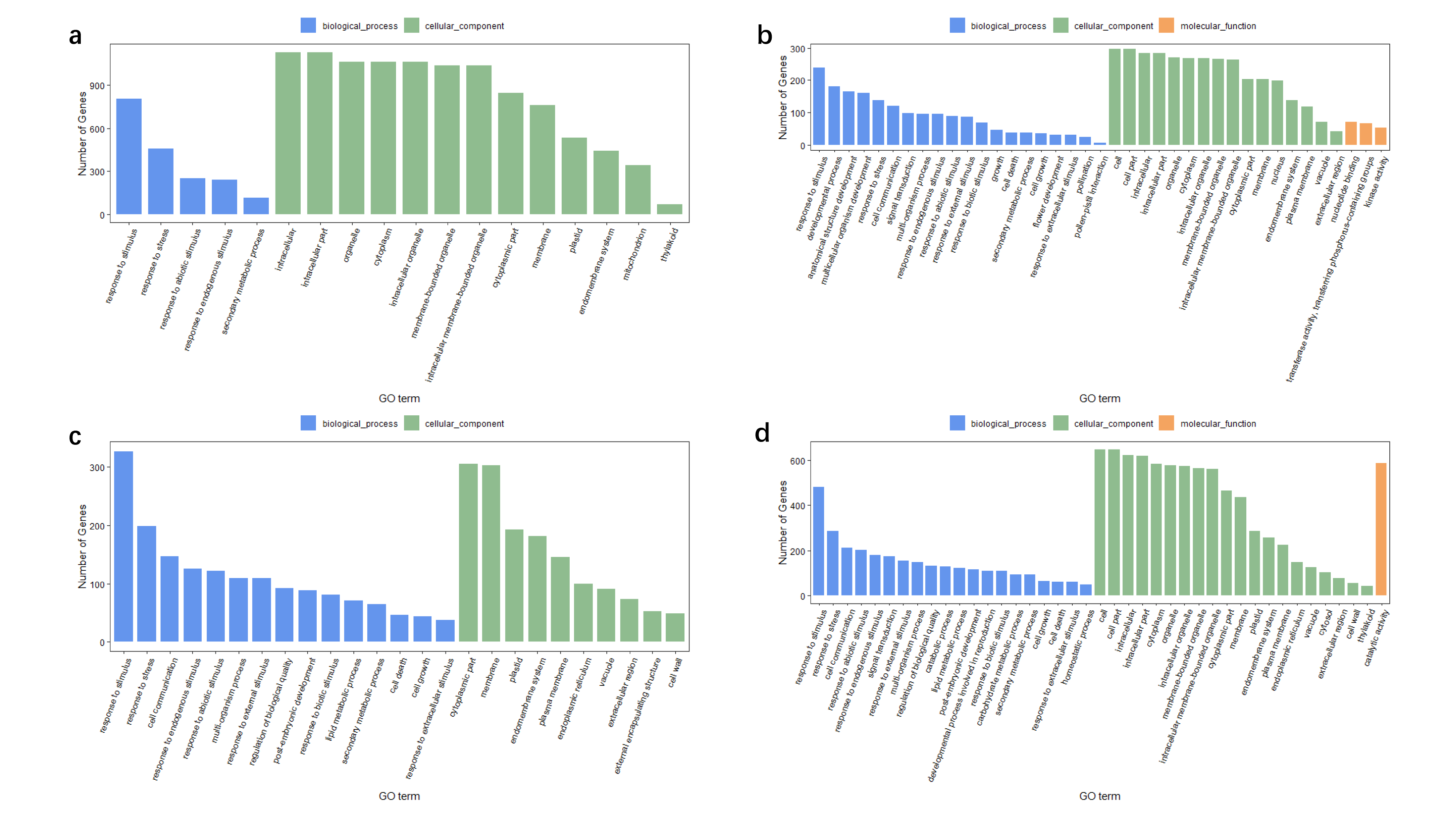

Supplement: Supplementary file 1 [file plants-10-02257-s001.zip › Figure S7.tif]

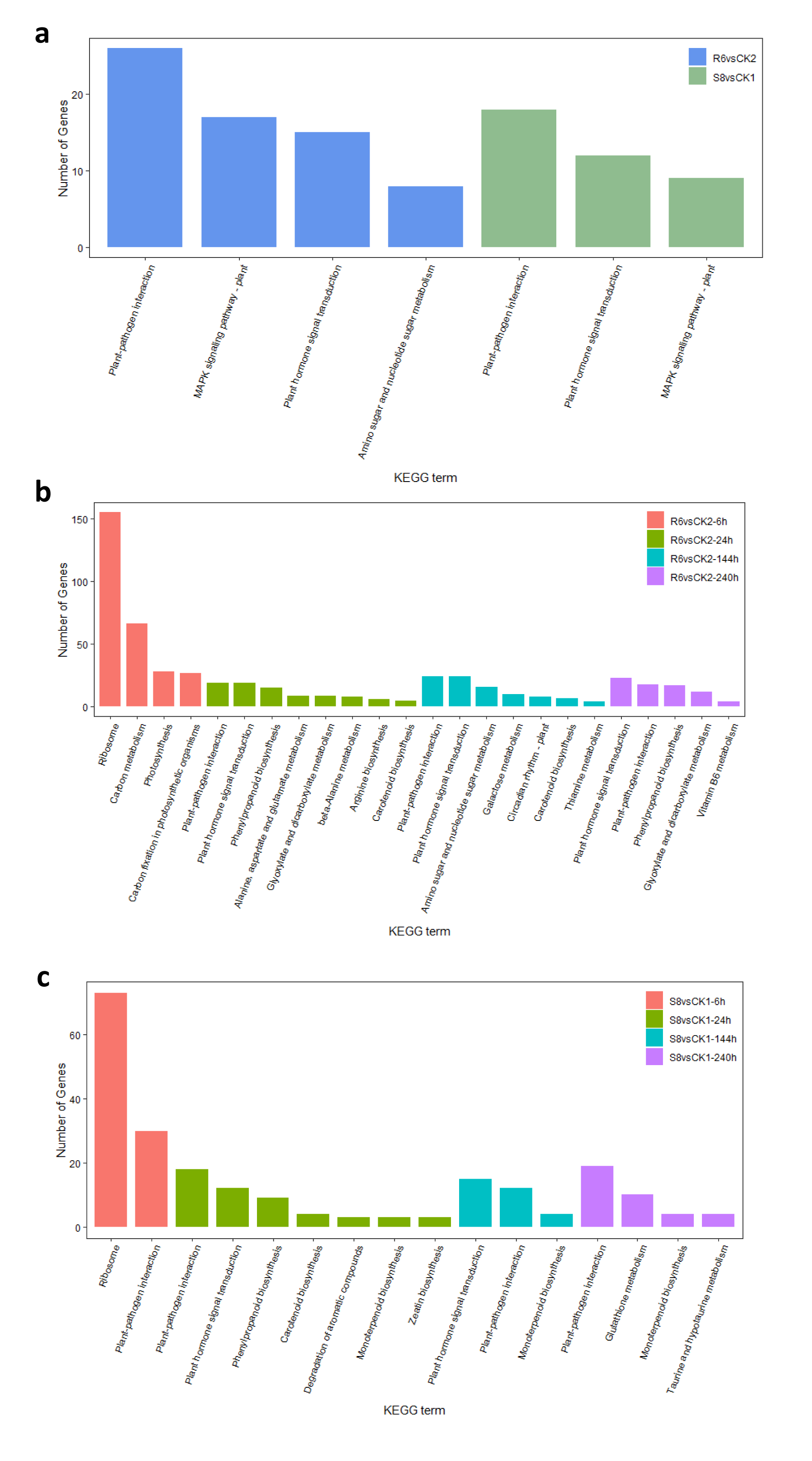

Supplement: Supplementary file 1 [file plants-10-02257-s001.zip › Figure S8.tif]

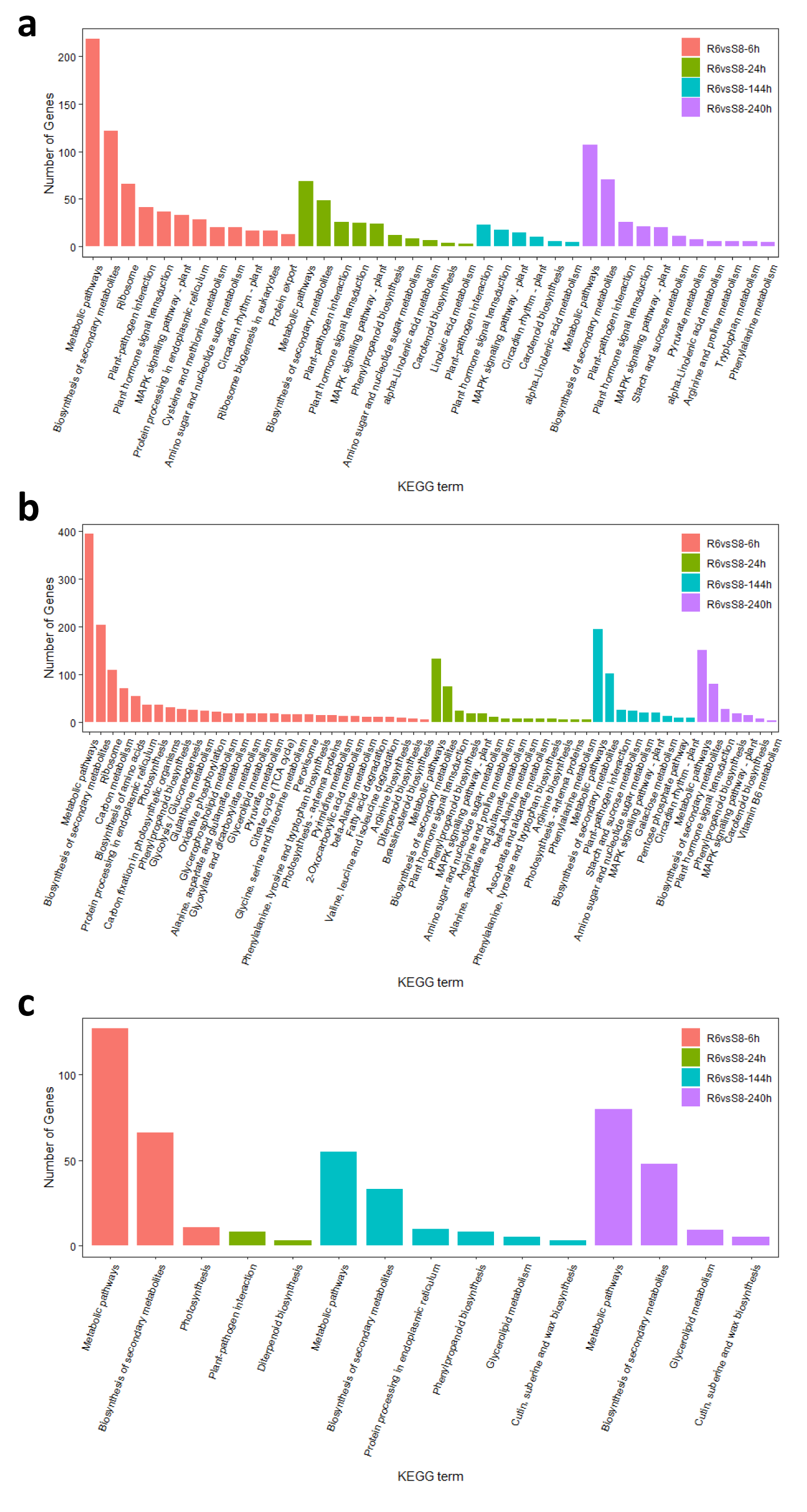

Supplement: Supplementary file 1 [file plants-10-02257-s001.zip › Figure S9.tif]
